# Hepatocyte-specific *Sox9* knockout ameliorates acute liver injury by suppressing SHP signaling and improving mitochondrial function

**DOI:** 10.1186/s13578-023-01104-5

**Published:** 2023-08-30

**Authors:** Dan Qin, Rui Wang, Jinwei Ji, Duo Wang, Yuanyuan Lu, Shiyao Cao, Yaqing Chen, Liqiang Wang, Xiangmei Chen, Lisheng Zhang

**Affiliations:** 1 College of Veterinary Medicine/College of Biomedicine and Health, Huazhong Agricultural University, Wuhan, 430070 Hubei China; 2 Department of Nephrology, Chinese PLA General Hospital, Chinese PLA Institute of Nephrology, State Key Laboratory of Kidney Diseases, National Clinical Research Center for Kidney Diseases, 28th Fuxing Road, Beijing, 100853 China

**Keywords:** *Sox9*, Acute liver injury, Hepatocyte, *SHP*, Mitochondria

## Abstract

**Background and Aims:**

Sex determining region Y related high-mobility group box protein 9 (Sox9) is expressed in a subset of hepatocytes, and it is important for chronic liver injury. However, the roles of Sox9^+^ hepatocytes in response to the acute liver injury and repair are poorly understood.

**Methods:**

In this study, we developed the mature hepatocyte-specific *Sox9* knockout mouse line and applied three acute liver injury models including PHx, CCl_4_ and hepatic ischemia reperfusion (IR). Huh-7 cells were subjected to treatment with hydrogen peroxide (H_2_O_2_) in order to induce cellular damage in an in vitro setting.

**Results:**

We found the positive effect of Sox9 deletion on acute liver injury repair. Small heterodimer partner (SHP) expression was highly suppressed in hepatocyte-specific *Sox9* deletion mouse liver, accompanied by less cell death and more cell proliferation. However, in mice with hepatocyte-specific *Sox9* deletion and SHP overexpression, we observed an opposite phenotype. In addition, the overexpression of *SOX9* in H_2_O_2_-treated Huh-7 cells resulted in an increase in cytoplasmic SHP accumulation, accompanied by a reduction of SHP in the nucleus. This led to impaired mitochondrial function and subsequent cell death. Notably, both the mitochondrial dysfunction and cell damage were reversed when *SHP* siRNA was employed, indicating the crucial role of SHP in mediating these effects. Furthermore, we found that Sox9, as a vital transcription factor, directly bound to *SHP* promoter to regulate *SHP* transcription.

**Conclusions:**

Overall, our findings unravel the mechanism by which hepatocyte-specific *Sox9* knockout ameliorates acute liver injury via suppressing SHP signaling and improving mitochondrial function. This study may provide a new treatment strategy for acute liver injury in future.

**Graphical Abstract:**

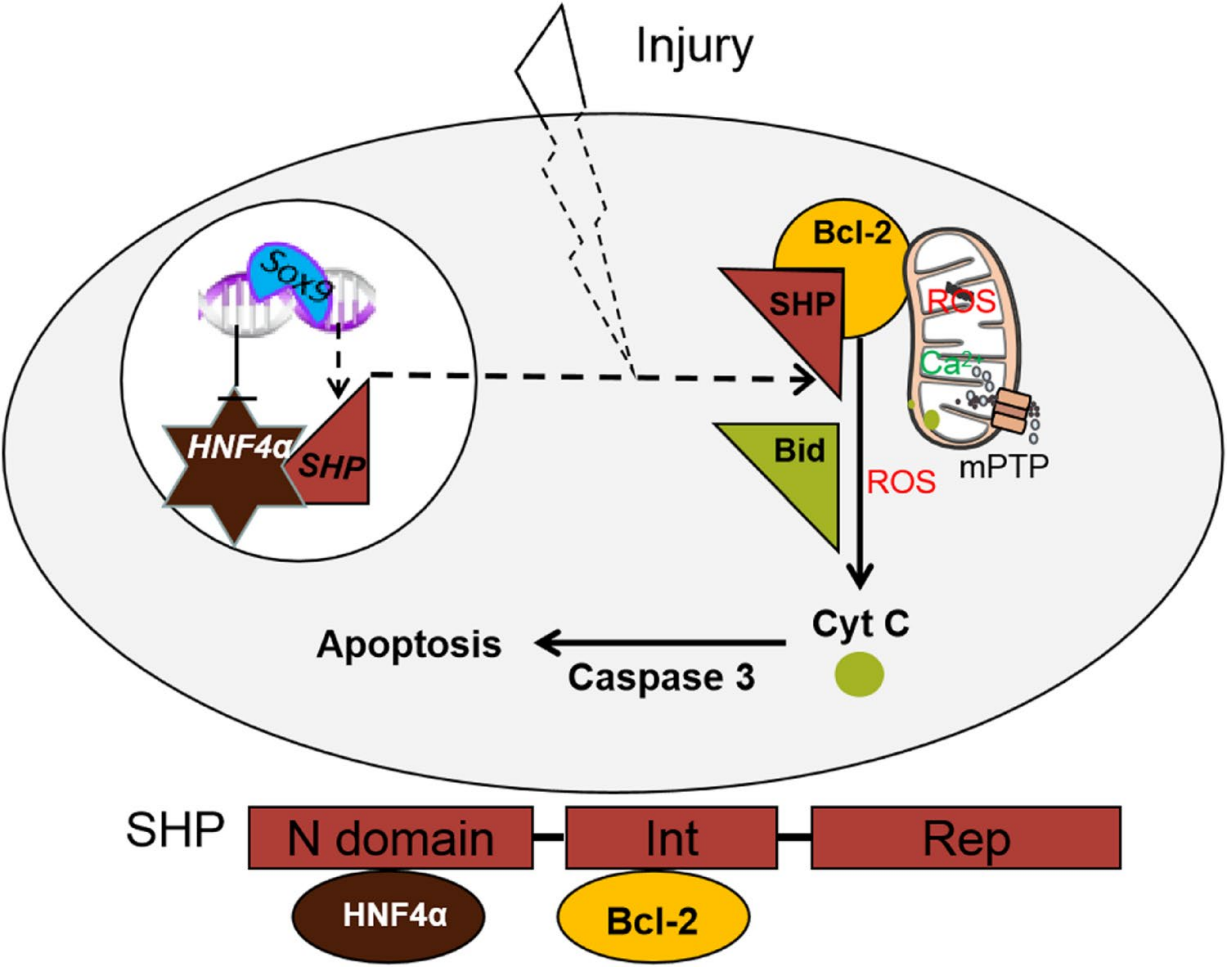

**Supplementary Information:**

The online version contains supplementary material available at 10.1186/s13578-023-01104-5.

## Background

Sox9 is the main transcription factor of Sox family, expressed in brain, gut, lung, kidney, liver and other tissues and organs [1–5]. In the liver, Sox9 is mainly expressed in biliary cells of both mice and humans, and thus it was once considered to be one of the most commonly used biliary markers [6–8]. Recently, Font-Burgada et al. have found that Sox9 is also expressed in a subset of hepatocytes, and *Sox9*^+^ hepatocytes are present around the ductal plate, and they are also known as hybrid hepatocytes (HybHP), because they jointly express cholangiocyte-specific marker CK19 and key hepatocyte marker HNF4α, and they proliferate extensively in response to chronic hepatocyte-depleting injuries [9]. Our previous studies indicate that *Sox9*^+^ hepatocytes can serve as bipotent progenitors to repair the liver after chronic liver injury [10, 11]. However, the role and mechanism underlying the *Sox9*^+^ hepatocytes in response to the acute liver injury have not been elucidated.

Acute liver injury and acute liver failure are syndromes characterized by rapid loss of functional hepatocytes in patients with no pre-existing signs of liver disease [12]. Ischaemia–reperfusion injury (IRI) is one of the typical acute liver injury during hepatectomy or transplantation. In the initial phase of liver IRI, the ischaemic injury exposes hepatocytes to hypoxia, dramatic pH changes, and ATP consumption, and increases their dependence on glycogen for energy production [13]. These events trigger the production of reactive oxygen species (ROS), augment intracellular calcium concentrations, and promote organelle damage, thus causing hepatic cell injury or death. IRI can trigger hepatocellular damage and inflammatory immune cascade reactions in the stressed liver, thus inducing multiple death pathways including apoptosis, necrosis, necroptosis, pyroptosis and ferroptosis [14, 15]. Increased generation of ROS has been identified as the main pathological mechanisms underlying IRI, and mitochondria contain major ROS generators including the respiratory chain components and numerous redox enzymes [14, 16, 17]. The subsequent reperfusion interferes with liver metabolism and elicits interconnected inflammatory cascade that exacerbates hepatocyte damage [14].

SHP, also termed as NR0B2, is an atypical orphan nuclear receptor and a death receptor, and it targets mitochondria to induce apoptosis [18, 19]. Structurally, SHP contains an N-terminal domain, an interaction domain, a repression domain, and it lacks a classical DNA binding domain, compared with other family members. Functionally, SHP is a pleiotropic receptor that plays a key role in multiple metabolic disorders [20], such as dysregulation of hepatic bile acid homeostasis [21–23] and lipid metabolism [24, 25]. Recent studies provide strong evidence that overexpression of SHP inhibits hepatocyte proliferation and activates hepatocyte apoptosis [13, 19]. Besides, it has been reported that SHP is situated in the outer membrane of mitochondria and has a domain that interacts with the transmembrane domain of Bcl-2 in mitochondria, thus inducing the release of cytochrome C (Cyt C) and causing cell death [15]. Additionally, the N-terminal domain of SHP can interact with HNF4α negatively regulated by Sox9 to promote SHP nuclear translocation [15, 26]. The analysis of the expression and cellular localization of SHP provides essential information on the fate of injured hepatocytes.

In this study, we examined the effect of Sox9 as a regulatory factor in hepatocytes on hepatic acute injury. Moreover, we revealed the regulatory mechanism by which *SHP* was involved in *Sox9*-mediated liver injury repair.

## Materials and methods

### Mouse models of acute liver injury

All the animal studies and procedures followed the guidelines for the care and use of laboratory animals of Huazhong Agricultural University. Male mice aged 6–8 weeks were used for all experiments. *Alb*umin (*Alb*)-Cre^ERT2^ mice were obtained from Biocytogen (B-CM-014) and *Sox9*^flox/flox^ (*Sox9* f/f) mice were brought from the Jackson Laboratory (013106; California). The primer sequences for mouse genotype identification were listed in Additional file 1: Table S1. *Alb*-Cre^ERT2^ and *Sox9* f/f mice were crossed to generate an *Alb*-Cre^ERT2+/−^; *Sox9*^flox+/−^ mice, then the heterozygous mice were backcrossed with *Sox9* f/f mice to obtain *Alb*-Cre^ERT2/+^, *Sox9* f/f mice with hepatocyte-specific *Sox9* gene knocked out (H-*Sox9* KO) under the induction of tamoxifen (T5648; 80 mg/kg body weight/day; Sigma-Aldrich) once a day for 5 days. For avoiding the side effect of tamoxifen, there is a 2-day waiting period between the final injection and treatment [27]. Hepatic IRI model was built referring to the previous described [28]. Briefly, mice were subjected to hepatic ischemia for 1 h, followed by 6 h of reperfusion. To compensate for SHP, pEGFP-N1-*SHP* plasmid was delivered into H-*Sox9* KO mice via hydrodynamic tail vein injection 7 days and 1 day before hepatic ischemia–reperfusion [29, 30]. Briefly, mice were rapidly injected (5–8 s) with 30 μg endotoxin-free plasmids diluted in Ringer solution in a total volume equal to 10% of their body weight [29, 30]. Partial hepatectomy (PHx) was performed on 6–8-week-old H-*Sox9* KO mice and their sex-matched *Sox9* f/f littermates. Acute toxic hepatic injury was induced by intraperitoneal injection of 2 mL/kg body weight of a 20% CCl_4_ solution in olive oil. A single dose of BrdU (B5002, Sigma) was injected intraperitoneally at 50 mg/kg animal weight 2 h before sacrifice [31]. Blood was collected for biochemical determination. Liver specimens were harvested at the indicated time points for fixation or cryopreservation.

### Cell culture and transfection

AML 12, Hepa1-6 and Huh-7 cells were maintained in H-DMEM containing 10% fetal bovine serum (FBS, Thermo Fisher). The *Sox9* gene was cloned in pcDNA 3.1 or pEGFP-N1 expression vector. siRNAs targeting *SHP* were obtained from Sangon Biotech and the forward sequences were: 5′-CGCCCUAUCAUUGGAGAUGUUTT-3' and the reverse sequences were: 5′-AACAUCUCCAAUGAUAGGGCGTT-3′ [32]. Transfection was performed by lipofectamine 2000 or lipofectamine 8000 according to the manufacturer’s instruction.

### Western blot

For total protein extraction, liver tissues or cell samples were lysed in RAPI lysis buffer (P0013, Beyotime) containing protease inhibitors (P8340, Sigma) according to the manufacturer’s instructions. Nuclear components were extracted according to Cytoplasmic and Nuclear Protein Extraction Kit (BB-36021, Bestbio). Isolation of mitochondrial proteins was performed using the Mitochondria Fractionation Kit (C3603, Beyotime Inst. Biotech). This study used the antibodies against the following proteins: SHP (PA5-102494, Invitrogene); GAPDH (60004-I-Ig, Proteintech); Sox9 (SC-166505), Bax (sc-7480), Cyt C (sc-13156), Bid (sc-373939) and Caspase-3 p17 (sc-271028) from Santa Cruz.

### Quantitative real-time PCR (qRT-PCR) and RNA-seq

Total RNA was extracted from frozen liver tissues or cell samples with the TRIzol reagent (Invitrogen) following the manufacturer’s protocol. The transcriptome RNA sequence and data analysis were carried out by BENAGEN. For cells, nuclear RNA isolation was performed as described with some modifications [33]. Briefly, cells cultured in 10 cm dishes were transfected with either vehicle or *Sox9* overexpression plasmid for 42 h, subsequently, they were subjected to treatment with 400 μM H_2_O_2_ for 6 h, then rinsed twice with ice-cold PBS and finally harvested in 1 mL ice-cold PBS by scraping and centrifuged at 1500 rcf for 5 min. For tissue samples, the liver samples should be ground up and centrifuged to get the cell pellets. Cell pellets were then re-suspended with 200 μL lysate and incubated on ice for 5 min. The lysate was centrifuged at 500 rcf for 3 min at 4 °C to pellet the nuclei, and the supernatant was the cytoplasmic fraction. Then RNA was obtained by standard procedure. For qRT-PCR, equal amounts of RNAs from different fractionations were reverse-transcribed into cDNAs with PrimeScriptTM RT Reagent Kit (Takara). 18S or U6 was used as cytosolic or nuclear endogenous control, respectively [34]. qRT-PCR was carried out using the MonAmp™ SYBR® Green qPCR Mix (Low ROX). The relative gene expression was calculated by the 2^−ΔΔCt^ method. The primer sequences were shown in Additional file 1: Table S2.

### Molecular cloning and cell-based luciferase reporter assay

Putative Sox9 binding sites in *SHP* promoter sequence were analyzed using an online algorithm (Nucleotide Blast). According to this prediction, the gene promoter fragments (position—1940 to + 40, −1540 to 40, relative to the transcription start site) were individually amplified by PCR using mouse genomic DNA. Afterwards, these fragments were individually inserted into the pGL3-basic plasmid. To estimate the firefly luciferase activity, the above plasmids together with the phRL-TK plasmid were individually co-transfected into Hepa1-6 cells using lipofectamine 2000 (Invitrogen) following the manual. After incubation for 6 h, the cells were cultured with fresh medium and collected 24 h later. Dual luciferase assay (Promega) was used to detect the luciferase activity by using a Fluoroskan Ascent FL (Thermo Scientific, USA). Co-transfected renilla luciferase control reporter vector was as internal reference. Promoter activity is the ratio of firefly luciferase activity to renilla luciferase activity.

### Histology and immunofluorescence

Liver samples were fixed in 10% v/v formalin/PBS, dehydrated, and embedded in paraffin. Liver sections were stained with H&E staining (C0105S, Beyotime) or subjected to immunochemistry staining. Histomorphology was observed using a light microscope. Immunofluorescence staining was performed to detect targets using antibodies: anti-Sox9 (AB5535, Millipore, RRID: AB_2239761), anti-SHP (A5411, Lifespan Biosciences, RRID: AB_592350), anti-HNF4α (Ab41898, Abcam), anti-CK19 (GB11197, Servicebio), and anti-BrdU (GB12051, Servicebio) at 4 °C overnight. After being incubated with fluorophore-conjugated secondary antibody (A-11034, A-21424, Invitrogen), slices were counterstained with DAPI (Ab104139, Invitrogen). Finally, the fluorescence staining results were observed by confocal microscope (LSM710, Carl Zeiss Microscopy GmbH).

### Chromatin immunoprecipitation (ChIP) assay

ChIP was performed using the ChIP assay (P2080S, Beyotime) according to the user manual. In brief, mouse livers were incubated with formaldehyde (1%, v/v) for 10 min at 37 °C to crosslink the nuclear proteins to DNA. Subsequently, livers were rinsed with ice-cold PBS and lysed in SDS lysis buffer followed by sonication and immunoprecipitation with the antibody against Sox9 (AB5535, Millipore). IgG antibody binding reaction was used as a negative control. The captured chromatin was eluted and un-crosslinked, and the DNA was recovered. The ChIP-isolated DNA was subjected to PCR and qPCR analyses using the primer pair spanning *SHP* promoter region. The primer sequences were shown in attached Additional file 1: Table S2.

### TUNEL analysis and ROS staining

The TdT-mediated digoxigenin-dUTP nick-end labeling (TUNEL) method was performed using commercially available in situ apoptosis detection kits (11684795910, Roche and G1502, Servicebio) following the manufacturer's protocol. ROS such as superoxide and hydrogen peroxide was detected traditionally by staining techniques according to ROS detection assay (BB-470516, Bestbio).

### Mitochondrial function assays

Mito Tracker Red CMXRos (C1049B) and Calcein/PI Cell Viability/Cytotoxicity Assay was brought from Beyotime (C2015S). X-rhod-1, AM was purchased from Thermo Fisher (X14210). All these tests were performed according to the kit’s instructions. Morphological analysis of liver mitochondrial by transmission electron microscopy (Servicebio).

### Biochemical tests

The alanine aminotransferase (ALT) and aspartate aminotransferase (AST) levels in the plasma were measured with assays purchased from Nanjing Jiancheng Bio-engineering Institute (C009-2 and C010-2). For hepatic total bile acid (TBA) measurement, frozen livers were homogenized in PBS. Supernatant was collected for entire cell lysates after centrifugation at 2 500 rpm for 10 min at 4 °C. The hepatic TBA level in the supernatant or plasma was evaluated with kit from the Nanjing Jiancheng Bio-engineering Institute (E003-2-1).

### Statistical analysis

Experimental data were showed as the mean ± SD. Unpaired t-test with Welch’s correction or ANOVA was used to calculate the p value. Statistical significance was set at P < 0.05 (*), P < 0.01 (**), P < 0.001 (***,#, +). All measurements were taken from multiple independent biological replicates as indicated in each figure legend and were carried out independently at least three times.

## Results

### Generation and identification of tamoxifen-inducible ***Alb***-Cre transgenic mouse line ***Alb***-Cre^ERT2/+^; ***Sox9*** f/f

**Fig. 1:**
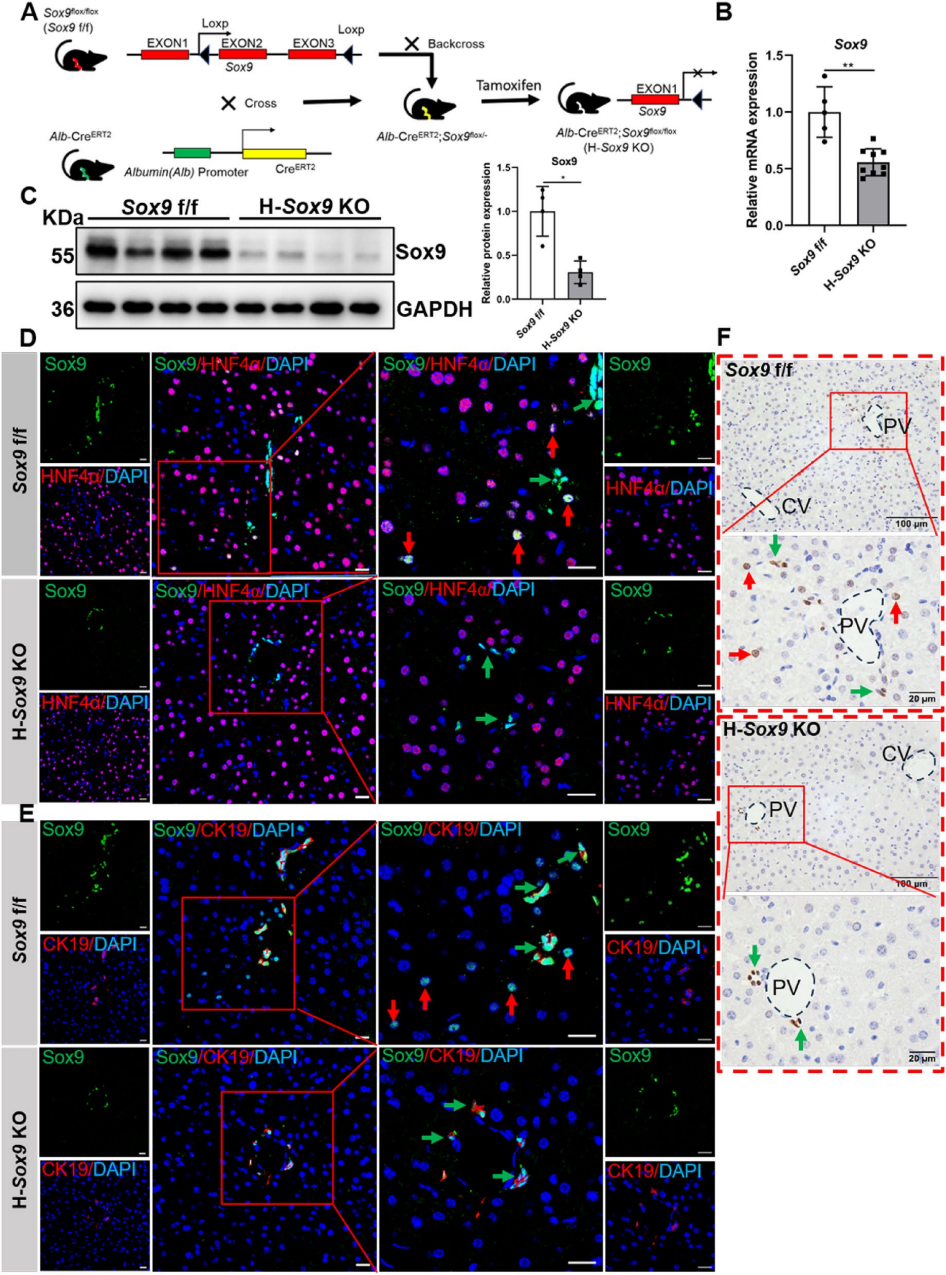
Generation and identification of the tamoxifen-inducible *Alb*-Cre transgenic mouse line *Alb*-Cre^ERT2/+^; *Sox9* f/f. **A** Schematic diagram showed the hepatocyte-specific *Sox9* knockout strategy. **B** Hepatic *Sox9* expression was determined by qRT-PCR in *Sox9* f/f mice (N = 5) and *Alb*-Cre^ERT2/+^; *Sox9* f/f mice (N = 9) treated with tamoxifen. **C** Western blot analysis of Sox9 protein level in liver tissues from *Sox9* f/f and H-*Sox9* KO mice (N = 4). **D** Sox9/HNF4α double staining was performed by immunostaining. Red arrows depict Sox9^+^ HNF4α^+^ cells and green arrows depict Sox9^+^ bile duct cells. Scale bar, 20 μm. **E** Sox9/CK19 double staining was performed by immunostaining. Red arrows depict Sox9^+^ hepatocytes and green arrows depict Sox9^+^ bile duct cells. Scale bar, 20 μm. **F** Representative Sox9 immunohistochemical staining of liver samples from *Sox9* f/f mice and H-*Sox9* KO mice. The red arrows indicate Sox9^+^HNF4α^+^ cells, while the green arrows indicate Sox9^+^ bile duct cells. PV: periportal vein; CV: central vein. Scale bar, 100 or 20 μm

Several studies have shown that Sox9^+^HNF4α^+^ hepatocytes are involved in chronic liver injury repair [35–37]. To assess the function of Sox9^+^ hepatocytes, the hepatocyte-specific *Sox9* gene knockout mouse line was established (Fig. 1A). Mouse genotypes were identified by PCR amplification and agarose gel electrophoresis (Additional file 1: Fig. S1A). Afterwards, adult *Alb*-Cre^ERT2/+^; *Sox9* f/f mice were injected with tamoxifen to generate H-*Sox9* KO mice. The Sox9 knockout efficiency was verified by qRT-PCR and western blot (Fig. 1B and C). To further verify the Sox9 knockout efficiency in hepatocytes, immunostaining assay was performed to investigate Sox9 expression in mouse livers. As shown in Fig. 1D–F, Sox9 was expressed both in bile duct cells and hepatocytes in *Sox9* f/f mouse livers, while in the H-*Sox9* KO mouse livers, Sox9 was only expressed in bile duct cells with no expression in hepatocytes. Additionally, no significant difference in AST and ALT levels was observed between *Sox9* f/f and H-*Sox9* KO mice, indicating the normal liver function of H-*Sox9* KO mice (Additional file 1: Fig. S1B). Taken together, our data demonstrated that the transgenic mouse line can achieve specific and temporal regulation of the deletion of the *Sox9* gene in hepatocytes.

### Hepatocyte-specific *Sox9* knockout ameliorates acute liver injury

**Fig. 2:**
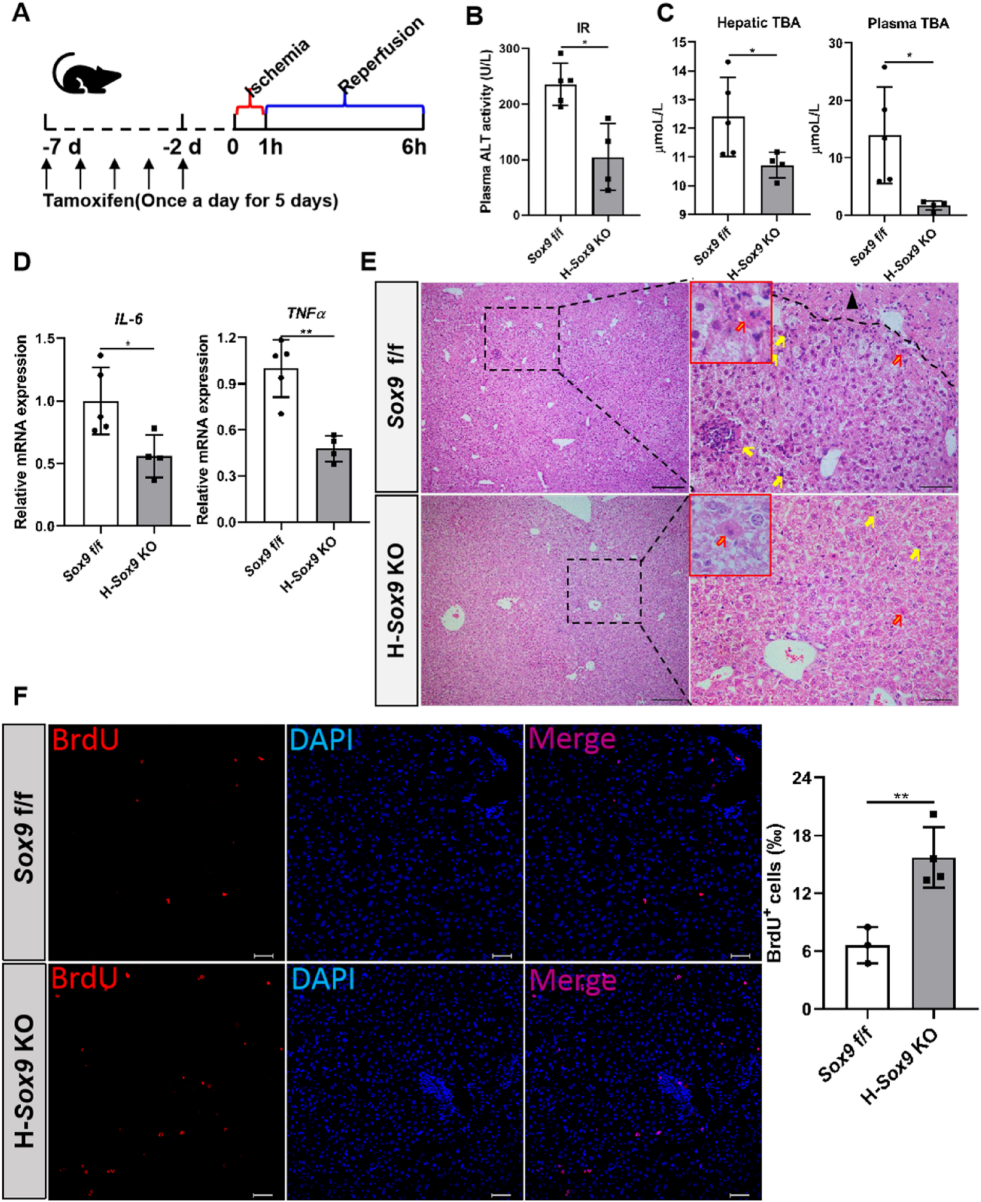
Hepatocyte-specific *Sox9* deletion ameliorates acute liver injury induced by IR. *Sox9* f/f and *Alb*-Cre^ERT2/+^; *Sox9* f/f mice pre-treated with tamoxifen were subjected to 1 h of hepatic ischemia. Plasma and liver samples were collected 6 h after reperfusion. **A** Schematic diagram showed ischemia reperfusion model. **B** Plasma ALT levels were measured in *Sox9* f/f and H-*Sox9* KO mice. N = 4 or 5. **C** TBA levels were detected in livers and plasma in *Sox9* f/f and H-*Sox9* KO mice. N = 4 or 5. **D** Levels of *IL-6* and *TNFα* mRNA expression were determined by qRT-PCR in *Sox9* f/f and H-*Sox9* KO mice. N = 4 or 5. **E** Representative liver sections stained with H&E. Yellow arrows indicate apoptotic cells (Red arrows indicate case prototypical apoptotic cells with higher magnification), while blank arrowhead indicates necrotic area. Scale bars: (left) 200 µm and (right) 50 µm. **F** Representative BrdU images in livers from *Sox9* f/f mice and H-*Sox9* KO mice induced by IR. Quantification of the percentage of BrdU^+^ cells in the indicated groups. Scale bar, 100 µm

In recent years, the role of Sox9^+^ hepatocytes in chronic liver injury repair has been widely documented [10, 38], but the role of Sox9^+^ hepatocytes in acute liver injury repair has rarely been reported. Hepatic IRI model was applied to investigate the function of Sox9^+^ hepatocytes in acute liver injury repair. As shown in Additional file 1: Fig. S2A and B, more Sox9^+^ hepatocytes appeared in IRI mouse liver. To examine the function of Sox9^+^ hepatocytes in acute liver injury, we constructed IRI model by using *Sox9* f/f and H-*Sox9* KO mice (Fig. 2A). Compared with control group, H-*Sox9* KO group exhibited the lower ALT and TBA levels, indicating that hepatic IR injury of H-*Sox9* KO mice was significantly ameliorated (Fig. 2B and C). As shown in Fig. 2D, qRT-PCR showed that the mRNA level of inflammation-related genes such as *IL-6* and *TNFα* was significantly decreased in H-*Sox9* KO mouse livers. H&E staining showed that IR led to inflammation, cell degeneration and apoptosis, and even tissue necroptosis in *Sox9* f/f mice, but hepatocyte-specific *Sox9* knockout contributed to acute liver injury repair (Fig. 2E). Furthermore, immunofluorescence assay was performed to determine the effects of hepatocyte-specific *Sox9* deletion on cell proliferation. As shown in Fig. 2F, BrdU-positive cells were increased in H-*Sox9* KO mouse livers, compared with those in the *Sox9* f/f group. Further, we investigated the role of Sox9^+^ hepatocytes in response to PHx and CCl_4_ acute liver injury, and the results were consistent with those of IRI (Additional file 1: Figs. S3 and S4). All the above results indicated that hepatocyte-specific *Sox9* deletion might alleviate acute liver injury by reducing inflammation and apoptosis and promoting cell proliferation.

### Sox9 directly regulates SHP in vivo and in vitro

**Fig. 3:**
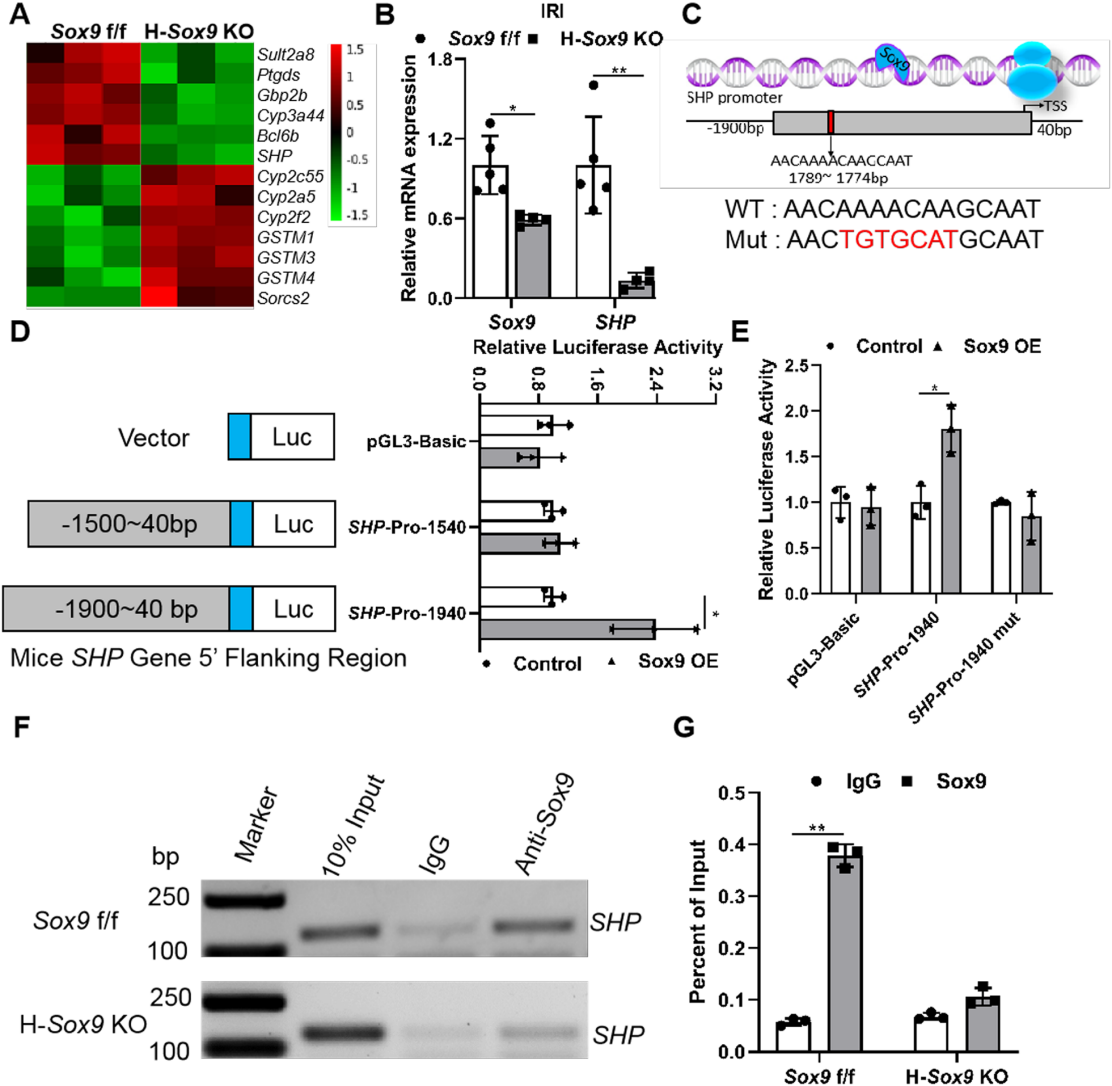
Sox9 enhances *SHP* transcription activity by binding to its promoter. **A** Heat map showed the differentially expressed genes in *Sox9* f/f and H-*Sox9* KO mice induced by IR. **B** Hepatic expression levels of *Sox9* and *SHP* in *Sox9* f/f and H-*Sox9* KO mice were determined by qRT-PCR analysis following IR injury. *36B4* was used as a housekeeping gene. N = 4–5 per group, data are shown as mean ± SD. **C** *SHP* promoter contained a putative Sox9 binding site. Sequences of the WT (black) and mutant (red) binding sites. **D** Amplification and activity detection of different fragments of *SHP* promoter (position 40 to − 1500, 40 to − 1900 relative to the transcription start site). These fragments were inserted into the pGL3-Basic vector and transfected into Hepa 1–6 cells with or without Sox9 expression plasmids and pRL-TK using lipofectamine 2000. N = 3. **E** *SHP*-promoter or *SHP*-mutation promoter was co-transfected into Hepa1-6 cells with pRL-TK for 24 h and samples were analyzed by dual-luciferase assays. N = 3 per group. The ratio of firefly luciferase activity to renilla luciferase activity in control group was set to 1. **F** ChIP analysis showed that Sox9 interacted with classic binding site on *SHP* promoter. **G** ChIP-qPCR assay of Sox9 level on *SHP* promoter using chromatin solutions prepared from *Sox9* f/f and H-*Sox9* KO mouse livers. N = 3

To explore the effects of Sox9^+^ hepatocytes on acute liver injury repair, RNA sequencing analysis of the livers of *Sox9* f/f and H-*Sox9* KO mice treated with IRI was performed [37]. The known Sox9 target gene Ptgds, multiple metabolic enzymes, and some new Sox9-related genes were identified by RNA-seq (Fig. 3A). Among these new genes, we focused on *SHP* since the down-regulation of *SHP* expression could improve liver pathological status [22, 39]. We validated the change in *SHP* gene expression at the mRNA level in IRI liver samples by qRT-PCR and immunofluorescence staining. The results showed that hepatocyte-specific *Sox9* knockout dramatically reduced *SHP* expression, which was consistent with the RNA-Seq results (Fig. 3B, Additional file 1: Fig. S5A). Besides, hepatocyte-specific knockout of *Sox9* significantly decreased *SHP* expression in the samples with liver injury induced by PHx and CCl_4_ (Additional file 1: Fig. S5B and C). To further investigate the regulation of SHP by Sox9, ectopic overexpression of Sox9 was performed in AML12, Hepa1-6, and Huh-7 cells. As shown in Additional file 1: Fig. S5D–F, Sox9 overexpression significantly induced *SHP* mRNA expression. These data indicate that hepatocyte-specific *Sox9* deletion might ameliorate IRI through *SHP* signaling.

### Sox9 regulates *SHP* transcription by binding to its promoter

Sox9 has a highly conserved HMG domain, and this domain recognizes and binds specific DNA sequences to alter target gene expression [40, 41]. Bioinformatics analysis shows that *SHP* promoter contains a sequence highly similar to the canonical Sox9 core motif AACAAT in the transcription region (Fig. 3C). In order to examine the impact of Sox9 on *SHP*, luciferase assay was performed to screen the possible binding sites. The results indicated that Sox9 overexpression increased *SHP* promoter activity, but promoter truncation abolished the increased activity (Fig. 3D). Likewise, site-directed mutagenesis of the binding element also abolished Sox9 overexpression-induced *SHP* promoter activity enhancement (Fig. 3E). Subsequently, ChIP assay demonstrated that Sox9 directly bound to the specific site of *SHP* promoter (Fig. 3F and G). Overall, Sox9 directly regulates *SHP *in vivo and in vitro.

### Hepatocyte-specific *Sox9* deletion promotes SHP accumulation in nucleus

**Fig. 4:**
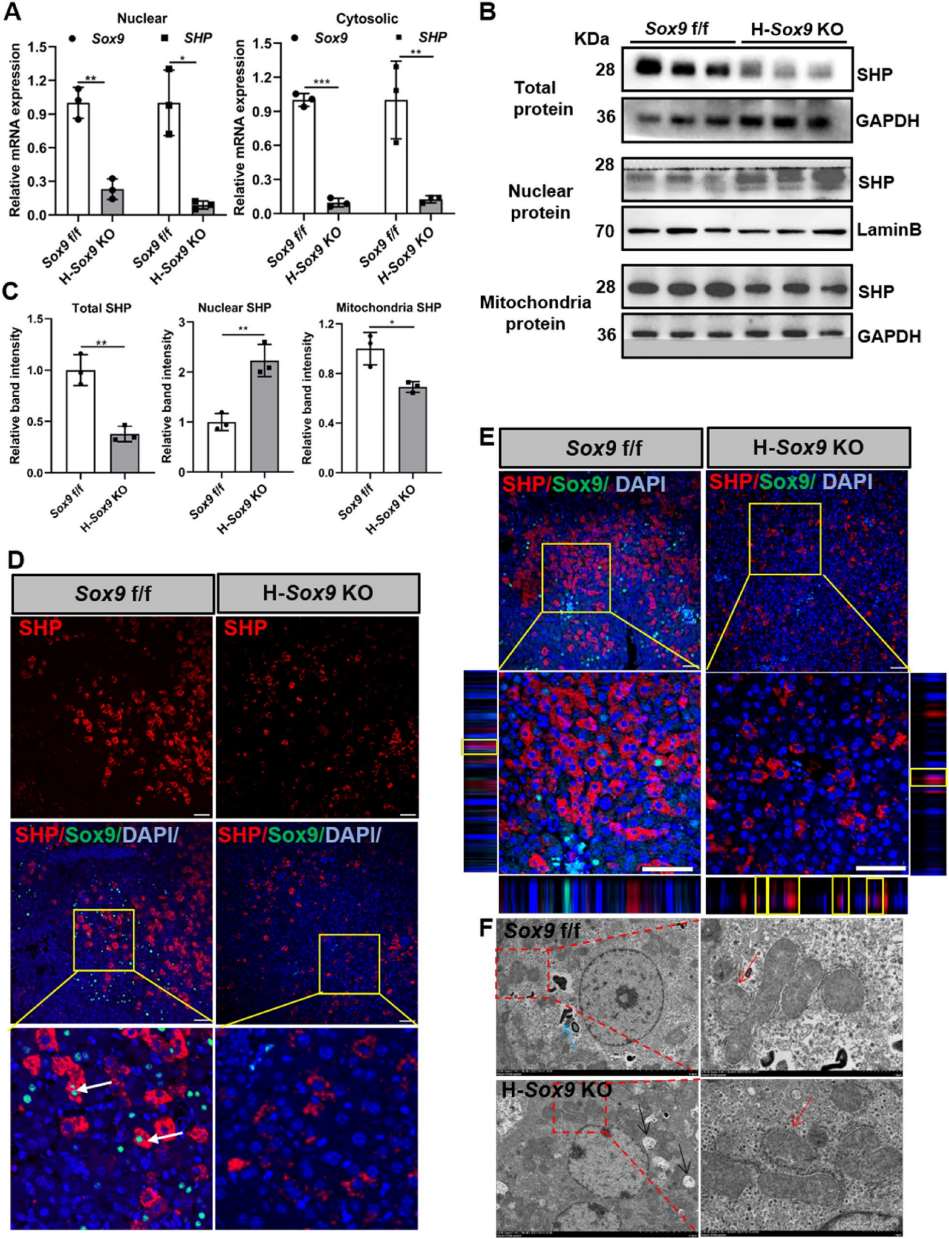
Hepatocyte-specific *Sox9* deletion promotes *SHP* accumulation in nucleus. *Sox9* f/f and *Alb*-Cre^ERT2/+^; *Sox9* f/f mice pre-treated with tamoxifen were subjected to 1 h of hepatic ischemia. Liver samples were collected 6 h after reperfusion. **A** qRT-PCR was used to determine *Sox9* and *SHP* mRNA in different cellular fractions in liver samples from *Sox9* f/f and H-*Sox9* KO mice. N = 3. **B** Western blot was used to determine SHP protein expression in different cellular fractions in liver samples. **C** Band intensities were measured by Image J. **D** Immunofluorescence co-staining for Sox9 and SHP. Scale bar, 50 µm. **E** The subcellular localization of SHP in hepatocytes was observed using confocal laser scanning microscopy. Scale bars, 50 µm. **F** Transmission electron microscopy analysis for hepatic mitochondrial morphology in *Sox9* f/f and H-*Sox9* KO mice induced by IR

To further decipher the mechanism by which hepatocyte-specific *Sox9* knockout alleviated IRI, we determined the cellular localization of SHP in liver samples, as it has been reported that SHP shuttles between the nucleus and the cytoplasm to regulate mitochondrial function [42–44]. The qRT-PCR results showed *SHP* mRNA levels in nuclear and cytoplasmic fractions were significantly decreased in hepatocyte-specific *Sox9* deletion liver (Fig. 4A). As shown in Fig. 4B and C, the expression of SHP protein was dramatically decreased in whole cell lysates, whereas SHP protein accumulation was significantly increased in the nuclear fraction, and it was significantly decreased in the mitochondrial fraction in H-*Sox9* KO mice relative to the *Sox9* f/f mice. The results of immunofluorescence detection were consistent with those of western blot detection. After ischemia-reperfusion injury, the expression of liver cytosolic SHP protein was significantly lower in H-*Sox9* KO mice than in control mice, but the expression of SHP nuclear protein was significantly higher (Fig. 4D and E). Moreover, the results of transmission electron microscopy showed that the knockout of hepatocyte-specific *Sox9* alleviated mitochondrial lesions, specifically, relieving the swelling of mitochondrial outer membrane and making the structure of mitochondrial ridge clearer in H-*Sox9* KO mice (Fig. 4F). Overall, our data suggest that loss of *Sox9* in hepatocytes may alleviate IRI by affecting SHP expression and cellular localization.

### Deletion of hepatocyte-specific *Sox9* inhibits IRI-induced cell death associated with SHP

**Fig. 5:**
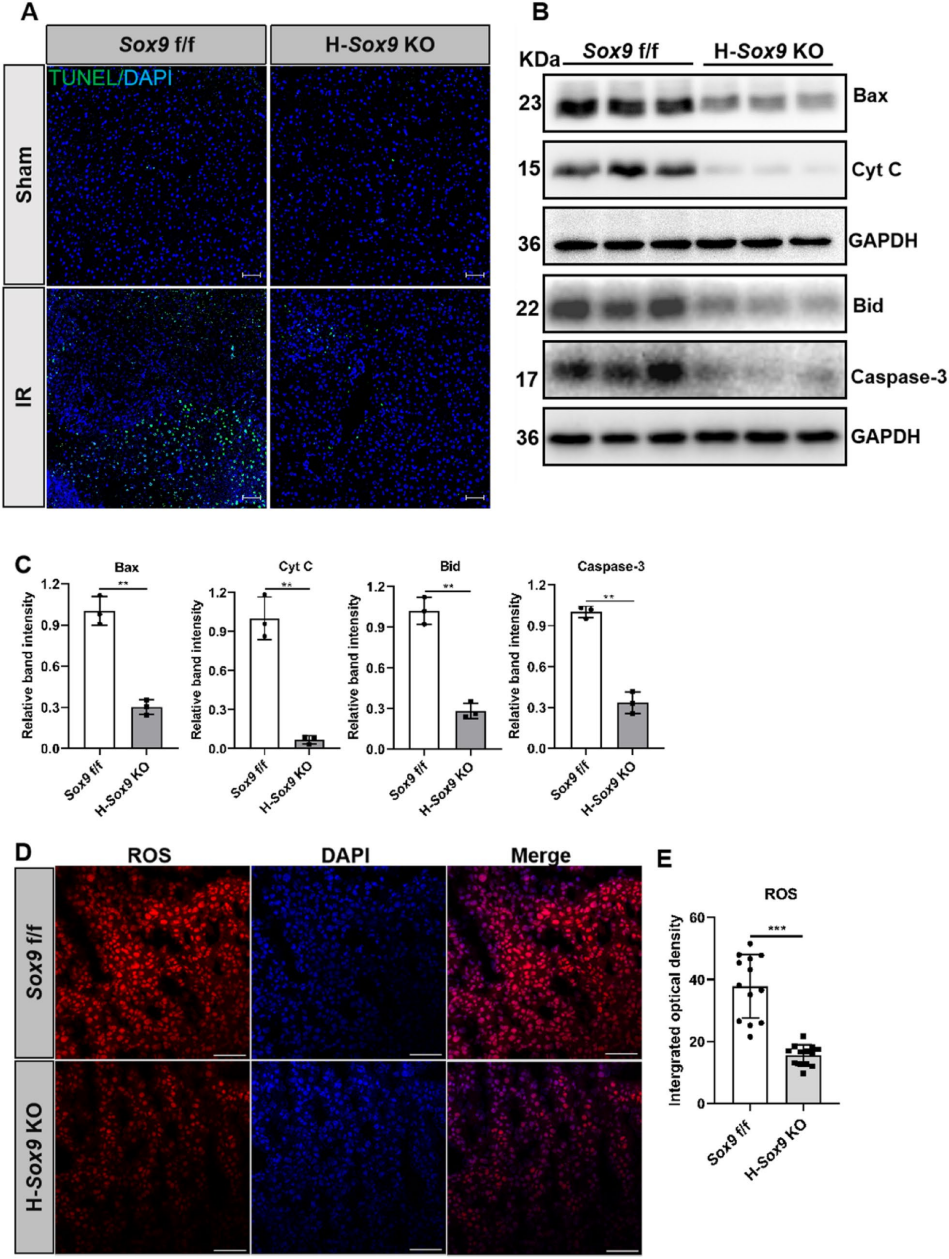
Loss of* Sox9* in hepatocytes inhibits IR-induced cell death associated with SHP. *Sox9* f/f and *Alb*-Cre^ERT2/+^; *Sox9* f/f mice pre-treated with tamoxifen were subjected to 1 h of hepatic ischemia, liver samples were collected 6 h after reperfusion. **A** The representative images of TUNEL staining in liver tissue sections from *Sox9* f/f and H-*Sox9* KO mice with or without IR. Scale bar, 50 µm. **B** Western blot analysis on the expression levels of apoptosis related proteins Bax, Cyt C, Bid and Caspase-3 p17 (Caspase-3) in liver tissues. GAPDH was used as an internal control. **C** Quantification analysis of apoptosis related proteins Bax, Cyt C, Bid and Caspase-3. N = 3. **D** ROS production in frozen liver tissues from *Sox9* f/f and H-*Sox9* KO mice following hepatic IR. Scale bar, 100 µm. **E** Quantitative ROS detection in liver samples

It has been reported that SHP is an active component of mitochondrial apoptosis [19]. Our above results demonstrated that hepatocyte-specific Sox9 knockout decreased SHP expression. To further evaluate the role of SHP in regulating apoptotic signaling in vivo, we performed TUNEL assays of liver sections from *Sox9* f/f and H-*Sox9* KO mice with or without IR. There were very few apoptotic cells in mice liver under steady state and IRI triggered robust apoptosis in *Sox9* f/f mice, whereas the number of apoptotic hepatocytes was dramatically decreased in H-*Sox9* KO mice (Fig. 5A). To investigate the regulatory effects of Sox9-SHP on mitochondrial apoptotic pathway, apoptosis-related proteins were analyzed. As shown in Fig. 5B and C, the expression level of pro-apoptotic protein Bax and Bid was markedly decreased in H-*Sox9* KO mice, and that of Cyt C and Caspase-3 was also significantly decreased in H-*Sox9* KO mice, thereby alleviating cell death. SHP has been reported to modulate mitochondrial respiration and ROS production [19]. In order to determine whether Sox9-mediated mitochondrial apoptosis was associated with liver oxygen consumption and energetics, the ROS was examined in liver samples of *Sox9* f/f mice or H-*Sox9* KO mice following hepatic IR. Compared to the control mice, hepatocyte-specific *Sox9* deletion mice exhibited the significantly reduced ROS (Fig. 5D and E). In conclusion, our results indicate that loss of *Sox9* in hepatocytes might alleviate IRI by inhibiting apoptosis-related protein accumulation to prevent cell death.

### Overexpression of *SOX9* reduces SHP nuclear translocation and induces cell death in vitro

**Fig. 6:**
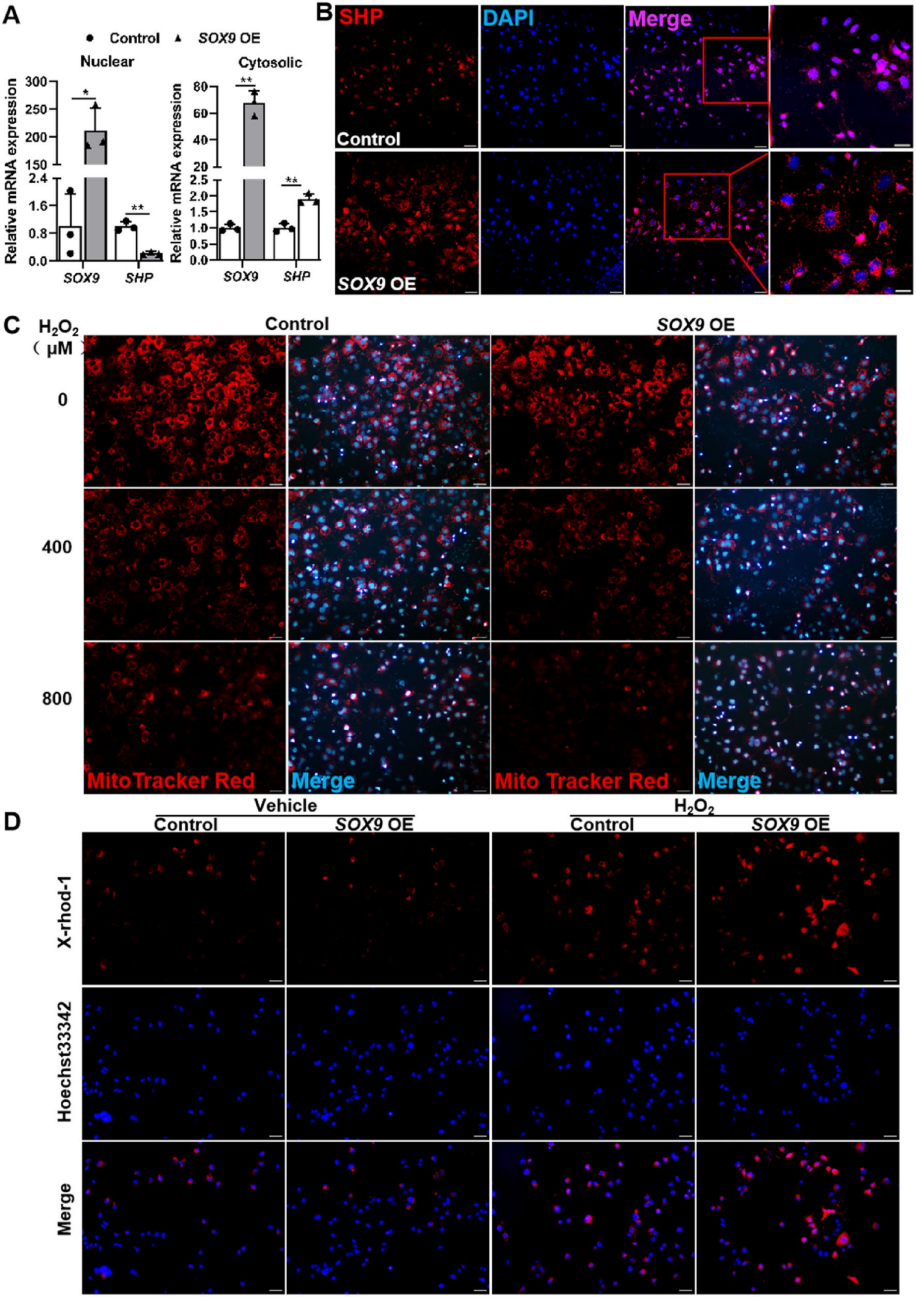
Overexpression of *SOX9* reduces SHP nuclear localization and induces mitochondrial injury. Huh-7 cells were transfected with either *SOX9* overexpression plasmid (*SOX9* OE) or vehicle plasmid (Control). Then the cells were treated with 400 μM H_2_O_2_ for 6 h before harvest. **A** qRT-PCR was used to determine* SOX9* and *SHP* mRNA expression in different cellular fractions in cell samples from indicated group. N = 3. **B** The subcellular localization of SHP in Huh-7 cells was observed under a fluorescence microscope. Scale bar, 50 µm. **C** Mito Tracker Red staining of Huh-7 cells treated with indicated concentration of H_2_O_2_. Scale bar, 50 µm. **D** Ca^2+^ indicator X-Rhod-1 in Huh-7 cells treated with vehicle or H_2_O_2_. Scale bar, 50 µm

To further verify the mechanism by which Sox9 regulated SHP nucleus-cytosolic shuttle, thus targeting mitochondria, eventually affecting cell death, *SOX9* was overexpressed in Huh-7 cells, and the Huh-7 cells were treated with H_2_O_2_ to induce cell damage. The results showed that both mRNA and protein levels of SHP were significantly decreased in nuclear fraction, but they were significantly increased in cytosolic fraction (Fig. 6A and B). We further examined whether *SOX9* overexpression could increase mitochondrial injury by membrane potential indicator or not. Mito Tracker Red CMXRos is a red-fluorescent dye staining mitochondria in live cells, and the red fluorescence accumulation is dependent upon membrane potential. X-Rhod-1, a special calcium ion indicator, can exhibit an increase in fluorescence intensity upon its binding to Ca^2+^. As shown in Fig. 6C and Additional file 1: Fig. S6A, H_2_O_2_ treatment reduced accumulation of Mito Tracker Red staining in Huh-7 cells, and *SOX9* overexpression further decreased this accumulation in Huh-7 cells. However, H_2_O_2_ treatment increased the accumulation of X-rhod-1 in Huh-7 cells, and the intensity of X-rhod-1 was further increased in Huh-7 cells upon *SOX9* overexpression, indicating that *SOX9* overexpression promoted Ca^2+^ influx in the presence of injury (Fig. 6D). The data in Fig. 6 and Additional file 1: Fig. S6B and C jointly suggested that *SOX9* overexpression caused cell apoptosis potentially by regulating SHP signal and targeting mitochondria.

### Hepatocyte-*Sox9* deletion protects against hepatic ischemia-reperfusion injury via a SHP-dependent manner

To determine whether the decreased membrane potential, increased Ca^2+^ influx and consequently cell death induced by *SOX9* overexpression were directly related to SHP, *SHP* siRNA was used to knock down *SHP* in Huh-7 cells which transfected with *SOX9* OE plasmids. As shown in Additional file 1: Fig. S7A and B, qRT-PCR results demonstrated that *SOX9* mRNA level was dramatically increased in *SOX9* OE group compared with vector group, while SHP expression was significantly reduced in *SHP* siRNA group compared with control group. And through the application of *SHP* siRNA, we observed a reversal of the heightened mitochondrial damage and cell death caused by SOX9 overexpression in the presence of H_2_O_2_ (Additional file 1: Fig. S7C, D and S8). These results suggest that *SOX9* overexpression-induced *SHP* upregulation is critical for cell death.

**Fig. 7:**
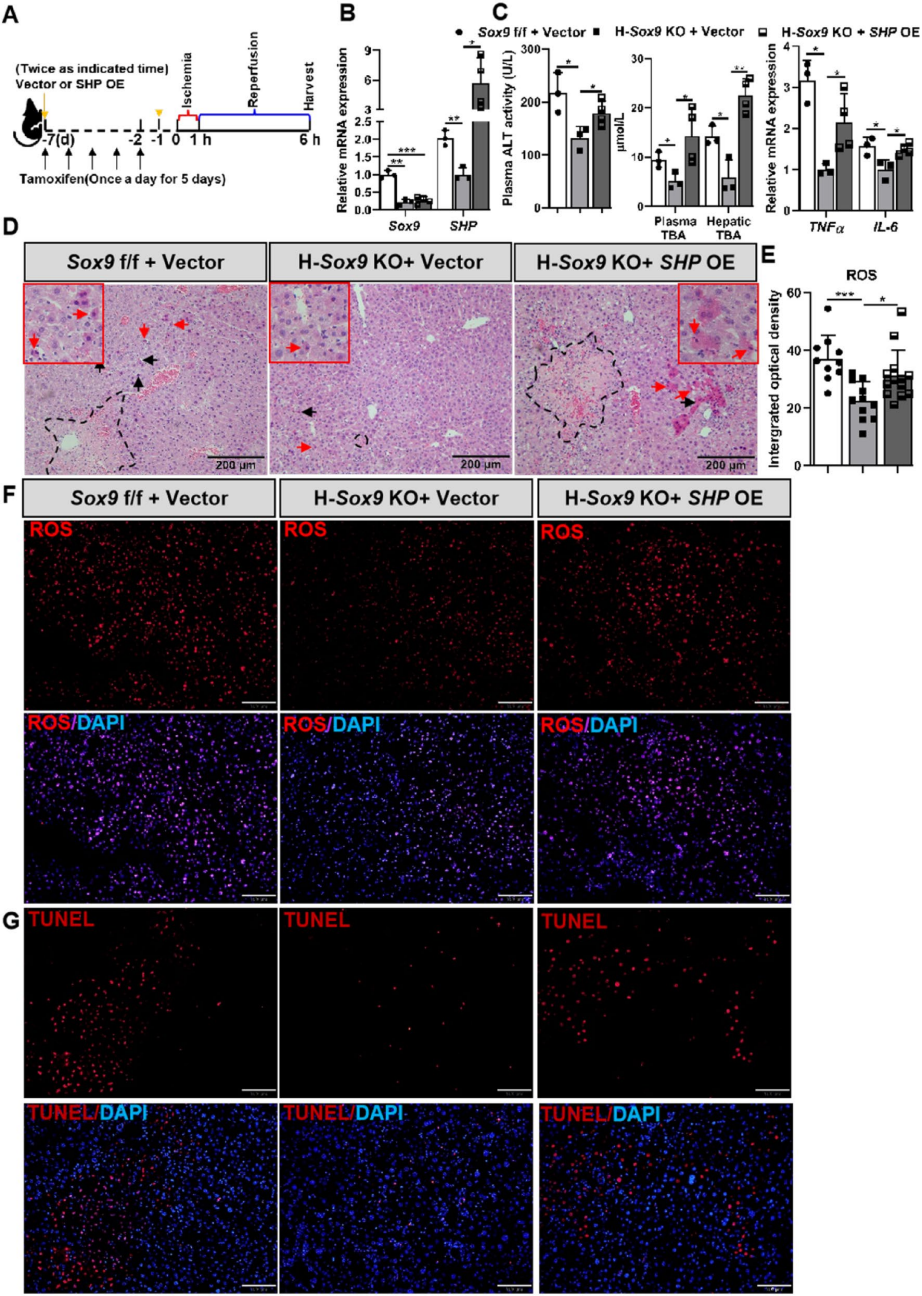
Overexpression of *SHP* aggravated hepatic ischemia–reperfusion injury in mice with hepatocellular specific knockout of *Sox9. Sox9* f/f and Alb-Cre^ERT2/+^; *Sox9* f/f mice pre-treated with tamoxifen and indicated vectors were subjected to 1 h of hepatic ischemia. Plasma and liver samples were collected at 6 h post reperfusion. **A** Schematic diagram showing ischemia reperfusion model. **B** *Sox9* and *SHP* mRNA expression were determined by qRT-PCR in indicated mice. **C** Plasma ALT level was measured in indicated group. TBA level was detected in livers and plasma in indicated group. *TNFα* and *IL-6* mRNA expression were determined by qRT-PCR in indicated group. **D** Representative liver sections stained with H&E. Arrows, cell apoptosis; Dashed box, necrotic area. Scale bar, 200 µm. **E** Quantitative ROS detection in liver samples. **F** ROS production in frozen liver tissues from *Sox9* f/f and H-*Sox9* KO mice following IR. Scale bar, 100 μm. **G** Representative liver sections stained with TUNEL. Scale bar, 100 μm

To further confirm the protective effect of *Sox9* KO against IR-induced liver injury might ascribe to the decreased *SHP*, H-*Sox9* KO mice were injected with either control vector or *SHP* overexpression (*SHP* OE) vector, followed by IRI (Fig. 7A). The *Sox9* knockout and *SHP* overexpression efficiency was verified by qRT-PCR (Fig. 7B). The inhibitory effects of hepatocyte-*Sox9* deficiency on liver damage, as evidenced by reduced levels of ALT and TBA, attenuation of the hepatic inflammatory response and decreased necrotic area, were markedly abolished by *SHP* overexpression (Fig. 7C and D). Furthermore, *SHP* overexpression reversed the protective effects of ROS and cell death observed in H-*Sox9* KO mice upon IRI (Fig. 7E–G). Collectively, these findings strongly suggest that SHP-mediated cell death contributed to the deleterious effects of *Sox9* in hepatic ischaemia-reperfusion injury.

In summary, our results indicate that hepatocyte-specific *Sox9* deletion induced *SHP* transcription silencing and SHP nuclear accumulation and consequently improved mitochondrial function, thereby contributing to the liver repair.

## Discussion

Sox9 plays an important role in the development of a variety of organs [45]. The formation of organs during normal development requires the precise activation and silencing of gene expression. The mutation of *Sox9* gene in embryonic stage can cause a rare and usually lethal syndrome CD (OMIM 114290) [45, 46], and *Sox9* homozygous mutant mice die at day 11.5 in embryonic stage, which seriously hampers the exploration for Sox9 functionality [6]. In this study, we developed an *Alb*-dependent conditional knockout mice with *Sox9* deleted in adult mouse hepatocytes by using Cre^ERT2^/LoxP recombinase system so as to explore Sox9 function. The mouse line successfully avoided the potential lesions caused by embryonic-stage *Sox9* deletion such as the delayed maturation of bile ducts, cholestasis, and the high levels of serum bilirubin [6, 47], and provided reference for studying the role of Sox9 in other organs.

Lineage tracing experiments show that Sox9^+^ hepatocyte is a sub-population of periportal hepatocytes, after chronic hepatocyte failure, this sub-population will experience extensive proliferation to replenish liver mass, which will not develop into hepatocellular carcinoma [7, 9]. After being transplanted into injured liver, the isolated Sox9^+^ hepatocytes display a great capacity to restore liver function, suggesting their therapeutic potential [7, 9, 36]. However, little is known about the role of Sox9^+^ hepatocytes in acute liver injury repair. Our data showed that the role of Sox9 in acute liver injury was different from that in chronic liver injury, and that hepatocyte-specific *Sox9* knockout alleviated acute liver injury induced by IR, CCl_4_, and PHx. Specifically, *Sox9* knockout in hepatocytes down-regulated the expression of pro-inflammatory cytokines *TNFα*, and *IL-6*, decreased TBA levels, inhibited cell apoptosis, and increased BrdU^+^ cell numbers. Our results are consistent with previous reports that Sox9 exacerbates primary hepatocyte damage by accelerating inflammation and apoptosis [38]. These findings suggest that *Sox9* knockout in hepatocytes with the aim to alleviate inflammation and promote cell proliferation might be effective strategy to reduce acute liver injury.

To explore the mechanisms by which *Sox9* deletion in hepatocytes promoted acute liver injury repair, we performed RNA-Seq to systematically analyze liver gene expression in *Sox9* f/f and H-*Sox9* KO mice following hepatic IR. The expression of most known genes related to metabolism altered after *Sox9* deletion. For example, Font-Burgada et al. have reported that the expression of genes related to oxidative drug metabolism were up regulated in hepatocytes that do not express *Sox9* [9]. Besides, our RNA-seq analysis identified several genes downregulated after *Sox9* knockout, including SHP. Previous studies have indicated that liver-specific *SHP* deletion prevents liver from fatty liver development and acetaminophen overdose-induced hepatotoxicity [39, 48]. Our cellular experiments showed that overexpression of *SOX9* in liver cell lines upregulated SHP expression and impaired mitochondrial function. Overall, the above findings suggest that *Sox9* depletion may attenuate acute liver injury by down-regulating SHP in adult hepatocytes. Besides, our luciferase reporter assay and ChIP assay results showed that Sox9 bound directly to the sequence from − 1789 bp to − 1774 bp in *SHP* transcriptional region to positively regulate *SHP* expression.

Numerous experiments conducted in vivo and in vitro have demonstrated that SHP is a critical component in mitochondrial apoptotic signaling pathway, and SHP regulates mitochondrial activity through its cellular localization [19]. SHP interacts with Bcl-2, thus destroying mitochondrial function, releasing Bid and Cyt C, activating Caspase-3, eventually inducing cell apoptosis. The interaction domain of SHP is mainly responsible for SHP mitochondrial localization by binding to the transmembrane domain of Bcl-2, and the N-terminal domain of SHP is critical for enhancing SHP nuclear translocation via HNF4α [15]. Notably, Sox9 has been reported to negatively regulate HNF4α [26]. In this study, we found that the knockout of *Sox9* in hepatocytes increased SHP nuclear translocation and decreased mitochondrial damage during IRI, and the overexpression of *SOX9* in Huh-7 cells treated with H_2_O_2_ decreased SHP expression in nuclear fraction but increased SHP expression in cytosolic fraction, which was accompanied by disrupted mitochondrial function. In addition, the inhibitory effects of hepatocyte-*Sox9* deficiency against liver injury or the negative effects of *SOX9* overexpression on cell damage was abolished by *SHP* overexpression or *SHP* siRNA, respectively. Our results are consistent with previous report [15]. Based on the above findings, it could be concluded that hepatocyte-specific *Sox9* deletion may regulate SHP nucleus-cytosolic shuttle, thus improving mitochondrial function, ultimately reducing acute liver injury.

## Conclusions

Sox9 has broad biological and physiological functions. Thus, it is vital to fully understand the biological and physiological properties of Sox9. This study reveals the molecular mechanism by which hepatocyte-specific *Sox9* deletion reduces *SHP* expression and increases SHP nuclear accumulation, thus inhibiting cell apoptosis, eventually ameliorating acute liver injury in mouse model. Therefore, targeting *Sox9* or blocking the *Sox9*-*SHP* axis may represent promising approaches to prevent or treat hepatic ischaemia–reperfusion injury.

### Supplementary Information

**Additional file 1: Figure S1.** Identification of tamoxifen-induced hepatocyte-specific *Sox9* deficient mice. (A) Genotyping by sequencing protocol. (B) Plasma AST and ALT levels were measured. N = 5 for *Sox9* f/f and N = 9 for H-*Sox9* KO group. **Figure S2.** Sox9 is overexpressed in IRI-induced mouse livers. Wild-type mice were subjected to 1 h of hepatic ischemia or sham surgery, liver samples were collected at 6 h post reperfusion. (A) *Sox9* mRNA level was measured (N = 4 per group). (B) Immunofluorescence co-staining for Sox9 and HNF4α. PV, periportal vein. Scale bar, 100 µm. **Figure S3.** Hepatocyte-specific *Sox9* knockout ameliorates PHx-induced acute liver injury. *Sox9* f/f and *Alb*-Cre^ERT2/+^; *Sox9* f/f mice pre-treated with tamoxifen were subjected to PHx, 48 h later, plasma and liver samples were collected for analysis. (A) Plasma ALT level was measured in *Sox9* f/f and H-*Sox9* KO mice. N = 4. (B) *TNFα* and *IL-6* mRNA expression were determined by qRT-PCR in * Sox9* f/f and H-*Sox9* KO mice. N = 4. (C) Representative liver sections stained with H&E. Scale bar, 100 µm. (D) Representative BrdU images in livers of *Sox9* f/f and H-*Sox9* KO mice induced by PHx. Scale bar, 100 µm. (E) Quantification of the percentage of BrdU^+^ cells in liver tissues. **Figure S4.** Hepatocyte-specific *Sox9* knockout ameliorates CCl_4_-induced acute liver injury. *Sox9* f/f and *Alb*-Cre^ERT2/+^; *Sox9* f/f mice pre-treated with tamoxifen were subjected to CCl_4_, 36 h later, plasma and liver samples were collected for analysis. (A) Plasma ALT level was measured in *Sox9* f/f and H*-Sox9* KO mice. N = 4–5. (B) *TNFα*, *IL-6* and *IL-1β* mRNA expression were determined by qRT-PCR in *Sox9* f/f mice and H-*Sox9* KO mice. N = 4–5. (C) Representative liver sections stained with H&E. Scale bar, 100 µm. (D) Representative BrdU images in livers of *Sox9* f/f and H-*Sox9* KO mice induced by CCl_4_. Scale bar, 100 µm. (E) Quantification of the percentage of BrdU^+^ cells in liver tissues. **Figure S5.** Loss of *Sox9* in hepatocytes decreases SHP expression, Sox9 overexpression increases SHP expression. (A) Representative confocal images of SHP (Red) and DAPI (blue) staining in livers. Scale bar, 50 μm. (B) Hepatic expression levels of *Sox9* and *SHP* in *Sox9* f/f and H-*Sox9* KO mice were determined by qRT-PCR analysis following PHx injury. *36B4* was used as a housekeeping gene. N = 4 per group. (C) Hepatic expression levels of *Sox9* and *SHP* in *Sox9* f/f and H-*Sox9* KO mice were determined by qRT-PCR analysis following CCl_4_ injury. *36B4* was used as a housekeeping gene. N = 4–5 per group. (D) Expression levels of *SOX9* and *SHP* in AML12 cells were determined by qRT-PCR analysis following *SOX9* overexpression (*SOX9* OE) or not. *GAPDH* was used as an internal control. (E) Expression levels of *Sox9* and *SHP* in Hepa1-6 cells were determined by qRT-PCR analysis following *Sox9* overexpression or not. *36B4* was used as an internal control. (F) Expression levels of *SOX9* and *SHP* in Huh-7 cells were determined by qRT-PCR analysis following *SOX9* overexpression or not. *GAPDH* was used as an internal control. **Figure S6.** Overexpression of *SOX9* induces cell death in Huh-7 cells treated with indicated concentration of H_2_O_2_. (A) Quantitative detection of Red Mito Tracker in Huh-7 cells (Corresponding to Fig. 6C). (B) Quantification of Calcein AM/PI staining in the control group or *SOX9* OE group treated with indicated concentration of H_2_O_2_. Calcein AM/PI double staining of Huh-7 cells. Compared with the control group, *SOX9* OE group resulted in increased cell death. Scale bar, 100 µm. **Figure S7.** Knocking-down of *SHP* reduces cell damage by protecting mitochondrial function. Huh-7 cells were transfected with either *SOX9* overexpression plasmid (*SOX9* OE), vector plasmid (Vector) or both; the latter condition involved co-transfection with siRNA targeting *SHP* followed by treatment with 400 μM H_2_O_2_ for 6 h before the harvest process. (A) Expression level of *SOX9* in Huh-7 cells was determined by qRT-PCR analysis. Student’s two-tailed t t-test (unpaired) was used to determine statistical significance differences between groups. Statistical significance was presented at the level of *P < 0.05. (B) Expression level of *SHP* in Huh-7 cells were determined by qRT-PCR analysis. ANOVA was used to determine statistical significance differences between groups. *p < 0.05 vs. Vehicle + *Control* siRNA; #p < 0.001 vs. Vehicle + *SHP* siRNA; + p < 0.001 vs. *Sox9* OE + *Control* siRNA. (C) Ca^2+^ indicator X-Rhod-1 in control and *SOX9* OE Huh-7 cells treated with H_2_O_2_. Scale bar, 50 µm. (D) Mito Tracker Red staining of control and *SOX9* OE Huh-7 cells treated with H_2_O_2_. Scale bar, 100 µm. **Figure S8.** Knocking-down of *SHP* mitigates cell death induced by *SOX9* overexpression in the presence of H_2_O_2_. Huh-7 cells were transfected with either *SOX9* overexpression plasmid (*SOX9* OE), vector plasmid (Vector) or both; the latter condition involved co-transfection with siRNA targeting *SHP* followed by treatment with 400 μM H_2_O_2_ for 6 h before the harvest process. Representative images displaying TUNEL staining in Huh-7 cells from the indicated experimental groups are presented with a scale bar of 200 µm. **Table S1.** The primer sequences for genotyping. **Table S2.** The primer sequences for qRT-PCR and ChIP.

## Data Availability

All data generated or analyzed during this study are included in this published article [and its supplementary information files].

## Abbreviations

Alb: Albumin
ALT: Alanine aminotransferase
AST: Aspartate aminotransferase
Cyt C: Cytochromes C
h: Hour
H&E: Hematoxylin and eosin
H_2_O_2_: Hydrogen peroxide
IR: Ischemia and reperfusion
IRI: Ischaemia–reperfusion injury
L: Litre
mg: Milligram
PHx: Partial hepatectomy
ROS: Reactive oxygen species
Sox9: Sex determining region Y related high-mobility group box protein 9
SHP: Small heterodimer partner
TBA: Total bile acid
TUNEL: TdT-mediated digoxigenin-dUTP nick-end labeling
