# Supplementary material for: Hepatocyte-specific Sox9 knockout ameliorates acute liver injury by suppressing SHP signaling and improving mitochondrial function
Source: Cell Biosci. 2023 Aug 30;13:159. doi: 10.1186/s13578-023-01104-5 (PMC10468867; doi:10.1186/s13578-023-01104-5)
Supplement: Supplementary file 1 — Additional file 1: Figure S1. Identification of tamoxifen-induced hepatocyte-specific Sox9 deficient mice. (A) Genotyping by sequencing protocol. (B) Plasma AST and ALT levels were measured. N = 5 for Sox9 f/f and N = 9 for H-Sox9 KO group. Figure S2. Sox9 is overexpressed in IRI-induced mouse livers. Wild-type mice were subjected to 1 h of hepatic ischemia or sham surgery, liver samples were collected at 6 h post reperfusion. (A) Sox9 mRNA level was measured (N = 4 per group). (B) Immunofluorescence co-staining for Sox9 and HNF4α. PV, periportal vein. Scale bar, 100 µm. Figure S3. Hepatocyte-specific Sox9 knockout ameliorates PHx-induced acute liver injury. Sox9 f/f and Alb-CreERT2/+; Sox9 f/f mice pre-treated with tamoxifen were subjected to PHx, 48 h later, plasma and liver samples were collected for analysis. (A) Plasma ALT level was measured in Sox9 f/f and H-Sox9 KO mice. N = 4. (B) TNFα and IL-6 mRNA expression were determined by qRT-PCR in Sox9 f/f and H-Sox9 KO mice. N = 4. (C) Representative liver sections stained with H&E. Scale bar, 100 µm. (D) Representative BrdU images in livers of Sox9 f/f and H-Sox9 KO mice induced by PHx. Scale bar, 100 µm. (E) Quantification of the percentage of BrdU+ cells in liver tissues. Figure S4. Hepatocyte-specific Sox9 knockout ameliorates CCl4-induced acute liver injury. Sox9 f/f and Alb-CreERT2/+; Sox9 f/f mice pre-treated with tamoxifen were subjected to CCl4, 36 h later, plasma and liver samples were collected for analysis. (A) Plasma ALT level was measured in Sox9 f/f and H-Sox9 KO mice. N = 4–5. (B) TNFα, IL-6 and IL-1β mRNA expression were determined by qRT-PCR in Sox9 f/f mice and H-Sox9 KO mice. N = 4–5. (C) Representative liver sections stained with H&E. Scale bar, 100 µm. (D) Representative BrdU images in livers of Sox9 f/f and H-Sox9 KO mice induced by CCl4. Scale bar, 100 µm. (E) Quantification of the percentage of BrdU+ cells in liver tissues. Figure S5. Loss of Sox9 in hepatocytes decreases SHP expression, So [file 13578_2023_1104_MOESM1_ESM.docx]

**Additional file**

**Additional Figures and Figure legends**

**
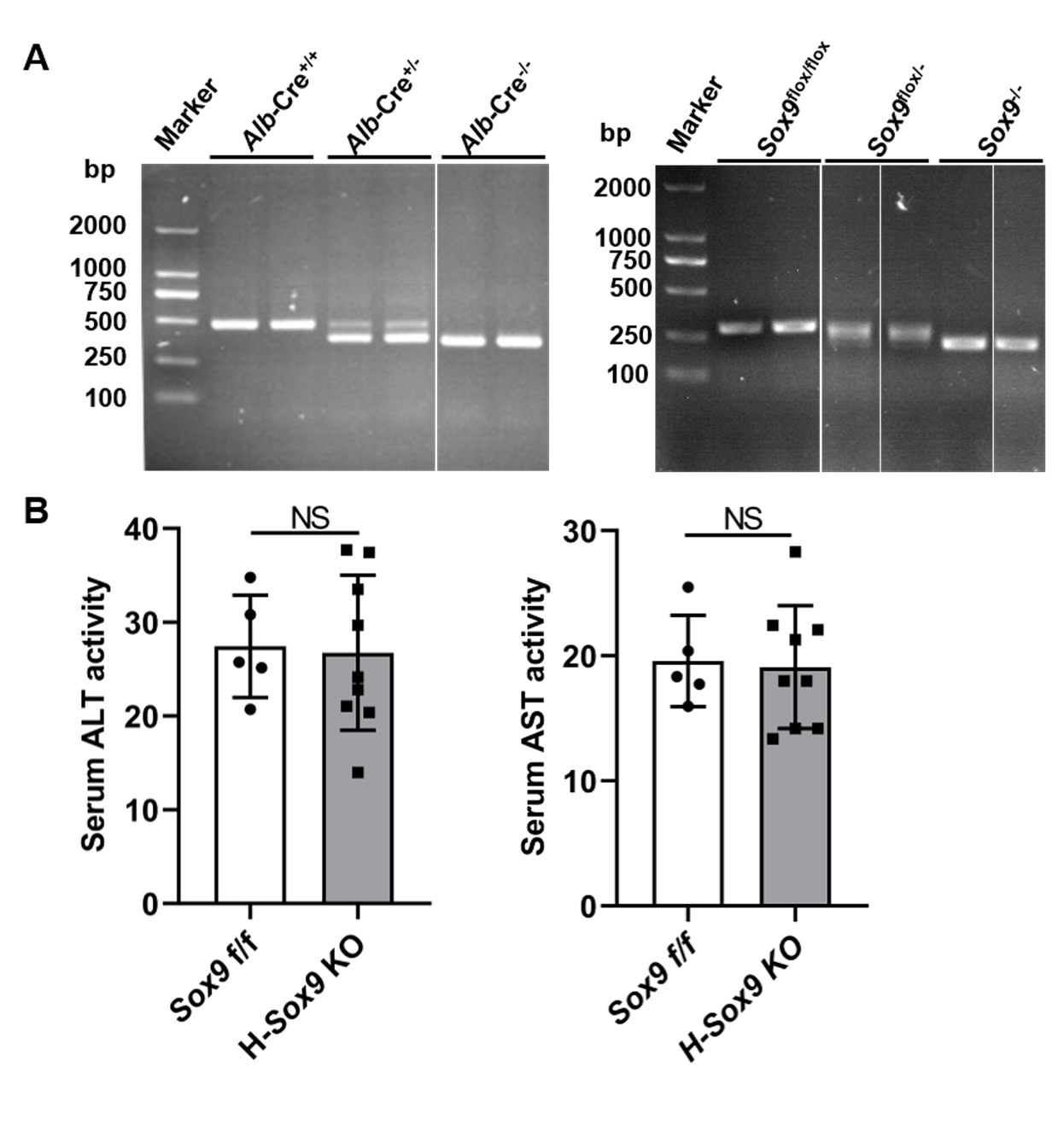
**

**Figure S1 Identification of tamoxifen-induced hepatocyte-specific *Sox9* deficient mice.**

(A) Genotyping by sequencing protocol.

(B) Plasma AST and ALT levels were measured. N=5 for *Sox9* f/f and N=9 for H-*Sox9* KO group.


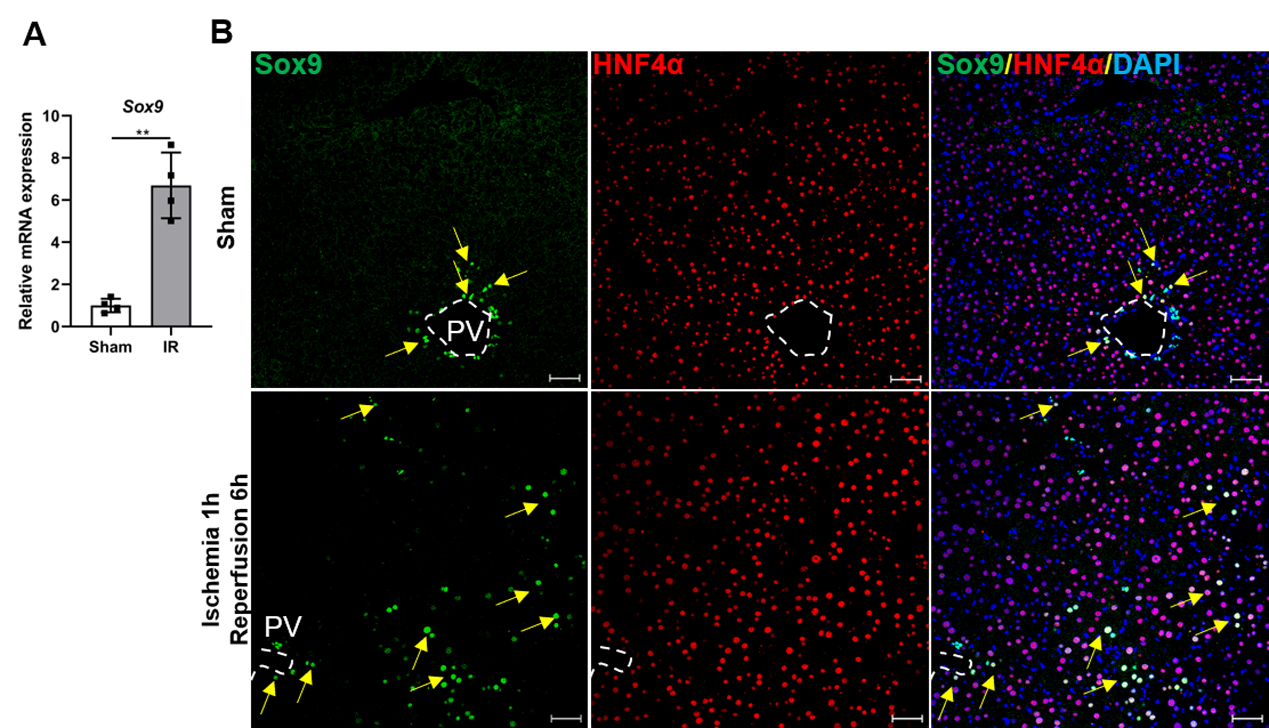


**Figure S2 Sox9 is overexpressed in IRI-induced mouse livers.**

Wild-type mice were subjected to 1 h of hepatic ischemia or sham surgery, liver samples were collected at 6 h post reperfusion.

1. *Sox9* mRNA level was measured (N=4 per group).
2. Immunofluorescence co-staining for Sox9 and HNF4α. PV, periportal vein. Scale bar, 100 µm.


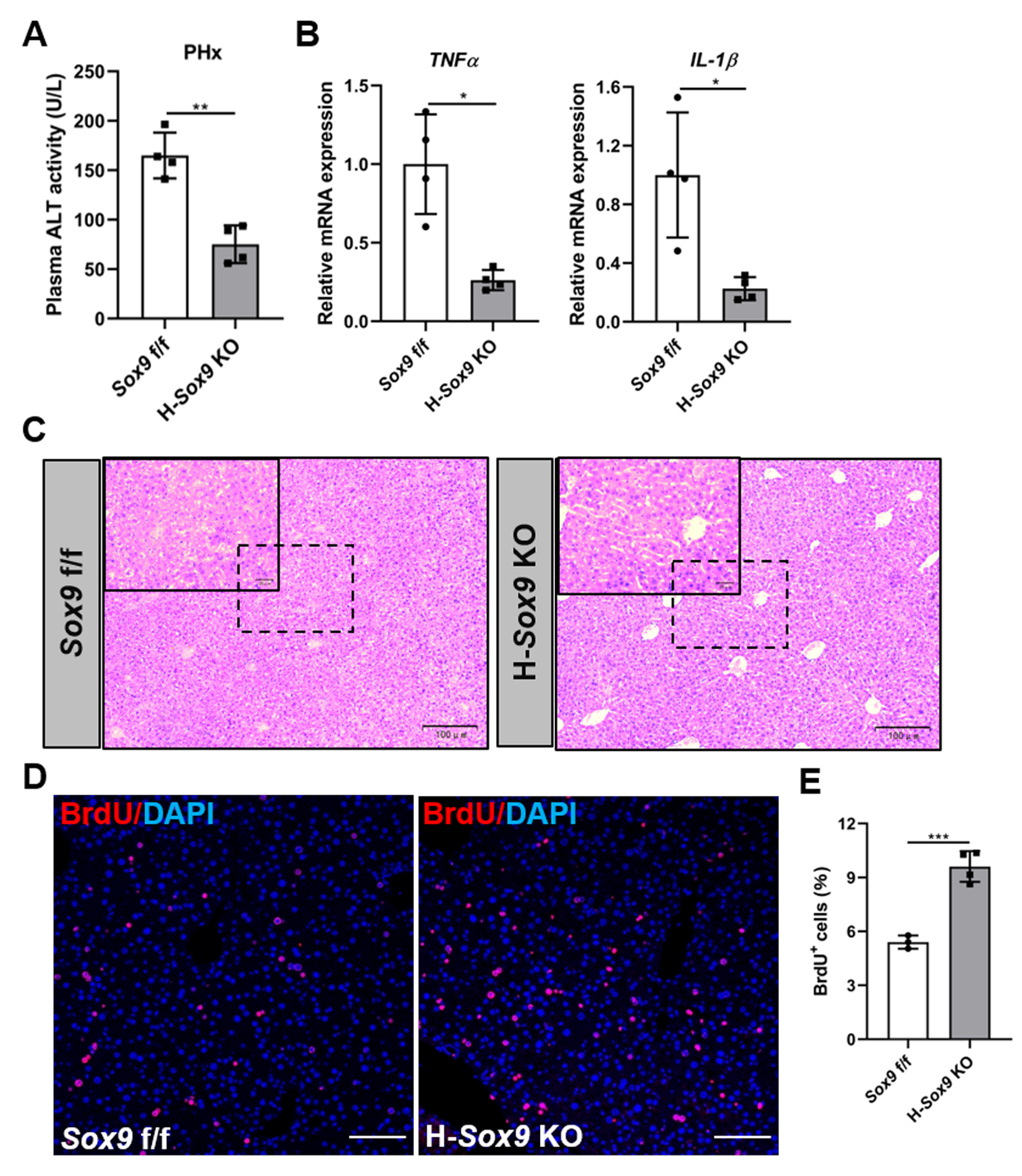


**Figure S3 Hepatocyte-specific *Sox9* knockout ameliorates PHx-induced acute liver injury.**

*Sox9* f/f and *Alb*-Cre^ERT2/+^; *Sox9* f/f mice pre-treated with tamoxifen were subjected to PHx, 48 h later, plasma and liver samples were collected for analysis.

1. Plasma ALT level was measured in *Sox9* f/f and H-*Sox9* KO mice. N=4.
2. *TNFα* and *IL-6* mRNA expression were determined by qRT-PCR in *Sox9* f/f and H-*Sox9* KO mice. N=4.
3. Representative liver sections stained with H&E. Scale bar, 100 µm.
4. Representative BrdU images in livers of *Sox9* f/f and H-*Sox9* KO mice induced by PHx. Scale bar, 100 µm.
5. Quantification of the percentage of BrdU^+^ cells in liver tissues.


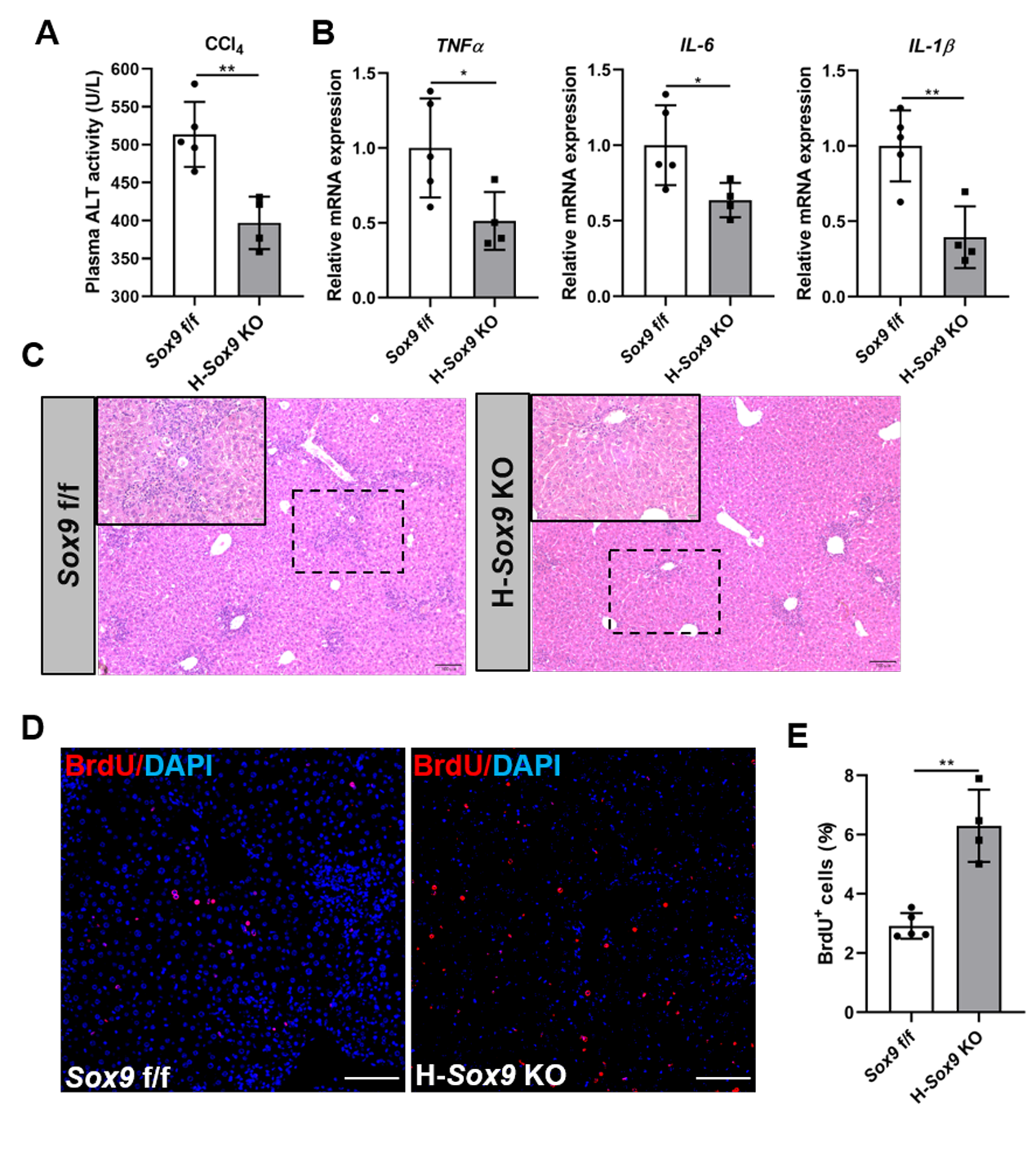


**Figure S4 Hepatocyte-specific *Sox9* knockout ameliorates CCl_4_-induced acute liver injury.**

*Sox9* f/f and *Alb*-Cre^ERT2/+^; *Sox9* f/f mice pre-treated with tamoxifen were subjected to CCl_4_, 36 h later, plasma and liver samples were collected for analysis.

(A) Plasma ALT level was measured in *Sox9* f/f and H*-Sox9* KO mice. N=4-5.

(B) *TNFα*, *IL-6* and *IL-1β* mRNA expression were determined by qRT-PCR in *Sox9* f/f mice and H-*Sox9* KO mice. N=4-5.

(C) Representative liver sections stained with H&E. Scale bar, 100 µm.

(D) Representative BrdU images in livers of *Sox9* f/f and H-*Sox9* KO mice induced by CCl_4_. Scale bar, 100 µm.

(E) Quantification of the percentage of BrdU^+^ cells in liver tissues.


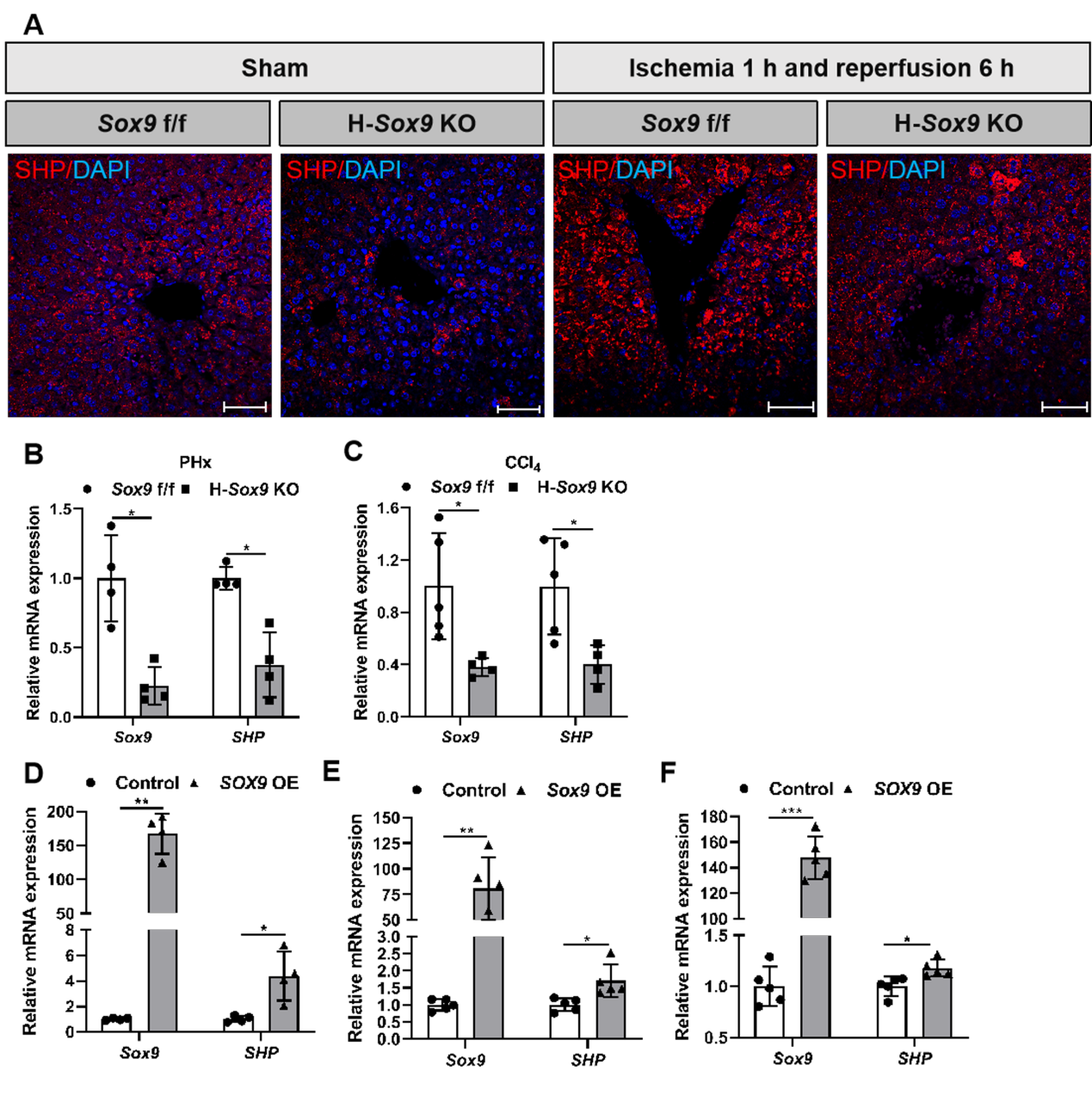


**Figure S5 Loss of *Sox9* in hepatocytes decreases SHP expression, Sox9 overexpression increases SHP expression.**

1. Representative confocal images of SHP (Red) and DAPI (blue) staining in livers. Scale bar, 50 μm.

(B) Hepatic expression levels of *Sox9* and *SHP* in *Sox9* f/f and H-*Sox9* KO mice were determined by qRT-PCR analysis following PHx injury. *36B4* was used as a housekeeping gene. N=4 per group.

(C) Hepatic expression levels of *Sox9* and *SHP* in *Sox9* f/f and H-*Sox9* KO mice were determined by qRT-PCR analysis following CCl_4_ injury. *36B4* was used as a housekeeping gene. N=4-5 per group.

(D) Expression levels of *SOX9* and *SHP* in AML12 cells were determined by qRT-PCR analysis following *SOX9* overexpression (*SOX9* OE) or not. *GAPDH* was used as an internal control.

(E) Expression levels of *Sox9* and *SHP* in Hepa1-6 cells were determined by qRT-PCR analysis following *Sox9* overexpression or not. *36B4* was used as an internal control.

(F) Expression levels of *SOX9* and *SHP* in Huh-7 cells were determined by qRT-PCR analysis following *SOX9* overexpression or not. *GAPDH* was used as an internal control.


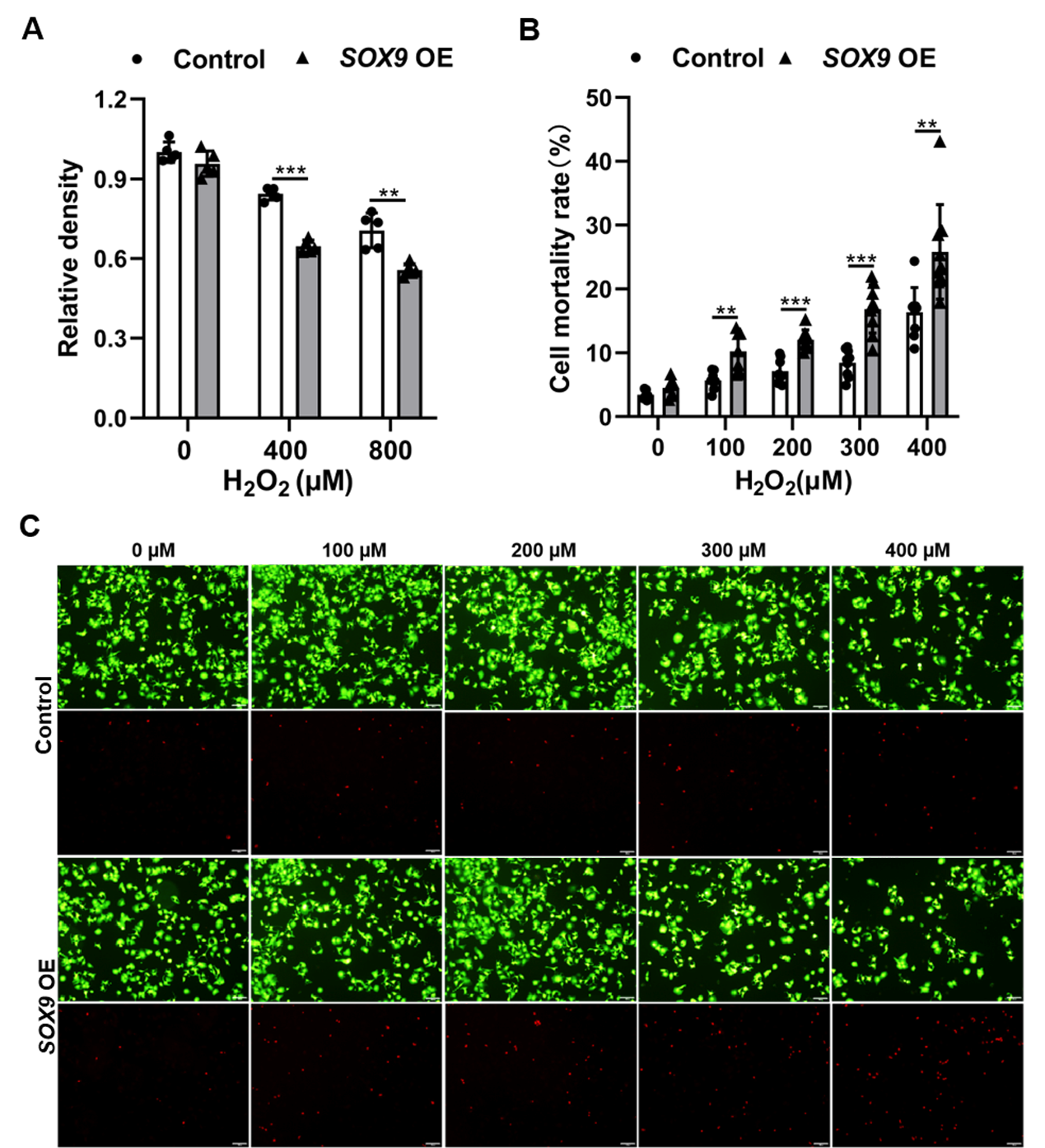


**Figure S6 Overexpression of *SOX9* induces cell death in Huh-7 cells treated with indicated concentration of H_2_O_2._**

1. Quantitative detection of Red Mito Tracker in Huh-7 cells (Corresponding to Figure 6C).
2. Quantification of Calcein AM/PI staining in the control group or *SOX9* OE group treated with indicated concentration of H_2_O_2_.
3. Calcein AM/PI double staining of Huh-7 cells. Compared with the control group, *SOX9* OE group resulted in increased cell death. Scale bar, 100 µm.

**
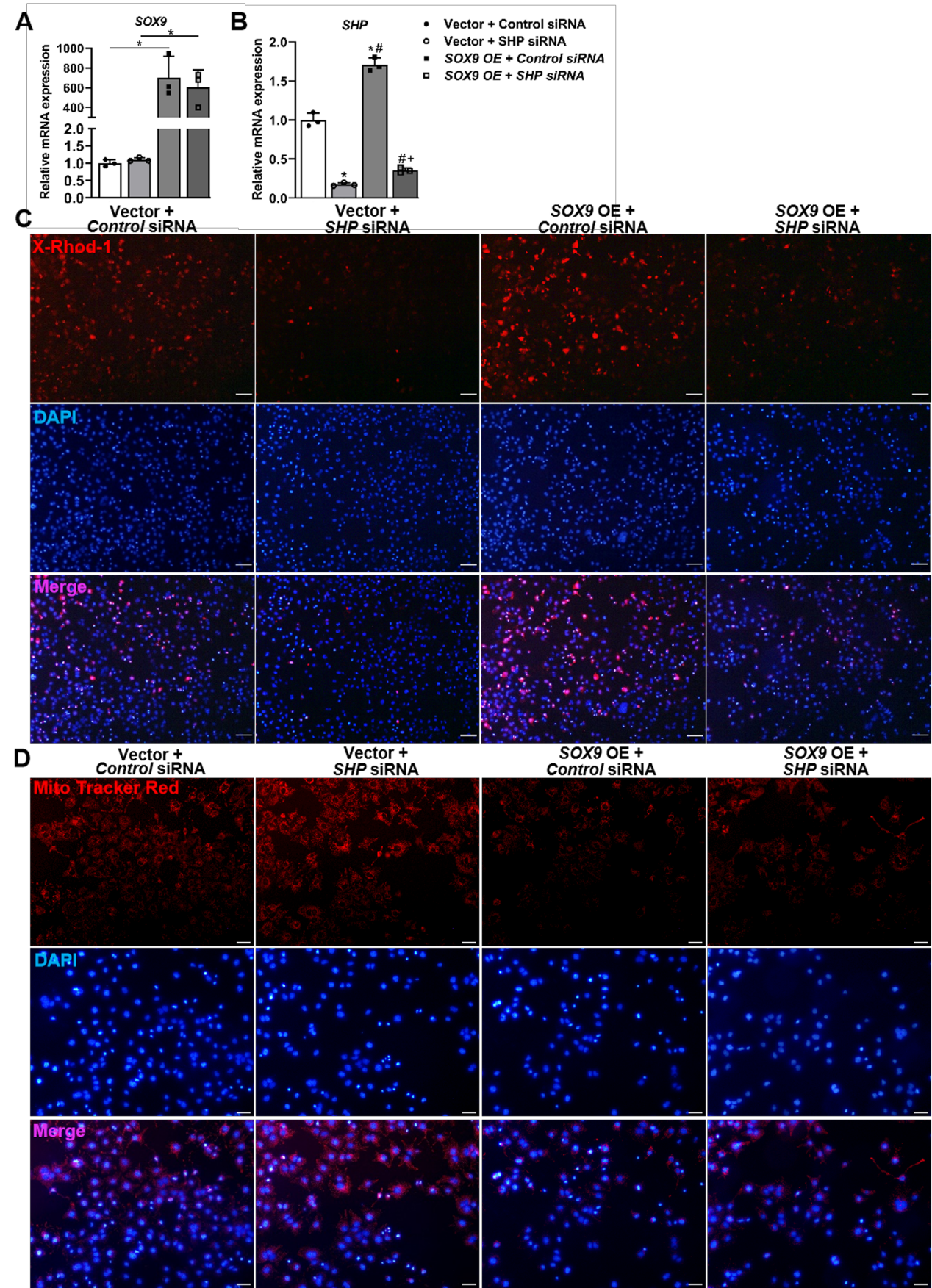
**

**Figure S7 Knocking-down of *SHP* reduces cell damage by protecting mitochondrial function.**

Huh-7 cells were transfected with either *SOX9* overexpression plasmid (*SOX9* OE), vector plasmid (Vector) or both; the latter condition involved co-transfection with siRNA targeting *SHP* followed by treatment with 400 μM H_2_O_2_ for 6 h before the harvest process.

(A) Expression level of *SOX9* in Huh-7 cells was determined by qRT-PCR analysis. Student’s two-tailed t t-test (unpaired) was used to determine statistical significance differences between groups. Statistical significance was presented at the level of *P<0.05.

(B) Expression level of *SHP* in Huh-7 cells were determined by qRT-PCR analysis. ANOVA was used to determine statistical significance differences between groups. *p<0.05 vs. Vehicle + *Control* siRNA; #p<0.001 vs. Vehicle + *SHP* siRNA; +p<0.001 vs. *Sox9* OE + *Control* siRNA.

(C) Ca^2+^ indicator X-Rhod-1 in control and *SOX9* OE Huh-7 cells treated with H_2_O_2_. Scale bar, 50 µm.

(D) Mito Tracker Red staining of control and *SOX9* OE Huh-7 cells treated with H_2_O_2_. Scale bar, 100 µm.


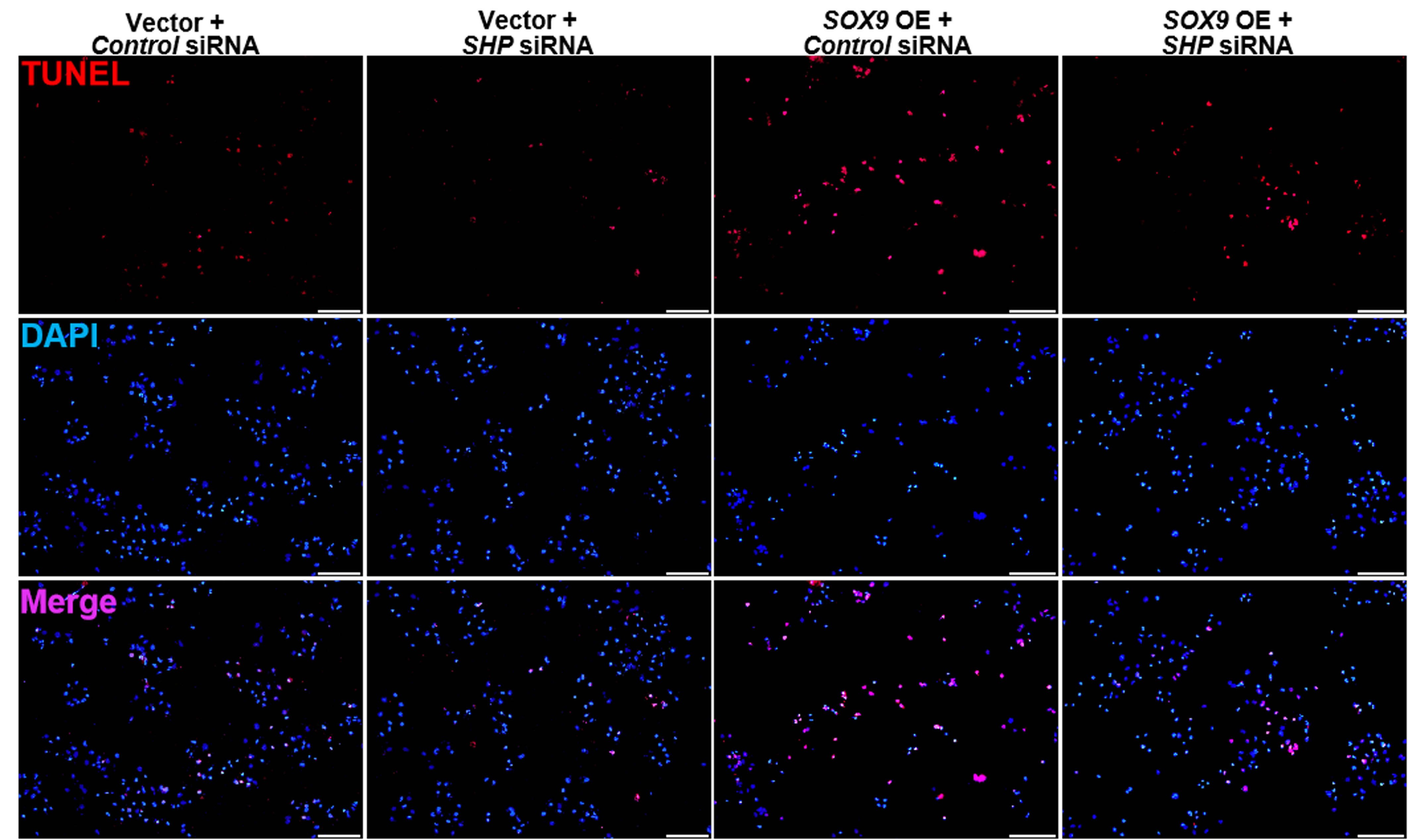


**Figure S8 Knocking-down of *SHP* mitigates cell death induced by *SOX9* overexpression in the presence of H_2_O_2_.**

Huh-7 cells were transfected with either *SOX9* overexpression plasmid (*SOX9* OE), vector plasmid (Vector) or both; the latter condition involved co-transfection with siRNA targeting *SHP* followed by treatment with 400 μM H_2_O_2_ for 6 h before the harvest process. Representative images displaying TUNEL staining in Huh-7 cells from the indicated experimental groups are presented with a scale bar of 200 µm.

**Supplemental Tables**

**Table S1. The primer sequences for genotyping**

| **Name** | **Sequence (5’-3’)** | **Length** |
| --- | --- | --- |
| *Sox9*^loxp^ F11576 | AGA CTC TGG GCA AGC TCT GG | WT:250bp  Mut:300bp |
| *Sox9*^loxp^ R11577 | GTC ATA TTC ACG CCC CCA TT |  |
| *Alb*-Cre^ERT2^  WT-F | TCTCCCCACCTCTAGCCCAAAGAAA | WT:385bp Mut:481bp |
| *Alb*-Cre^ERT2^  WT-R | ATCCCTCTAGGGCCTCAGGTTCTCA |  |
| *Alb*-Cre^ERT2^  Mut-F | GCATCGATACCGTCGACCTC |  |

**Table S2. The primer sequences for qRT-PCR and ChIP**

| Name | Application | Sequence (5’-3’) |
| --- | --- | --- |
| H-*GAPDH*-F | qRT-PCR | CTCTGGTAAAGTGGATATTG |
| H-*GAPDH*-R | qRT-PCR | GGTGGAATCATATTGGAAC |
| H-*Sox9*-F | qRT-PCR | ATGAATCTCCTGGACCCCTT |
| H-*Sox9*-R | qRT-PCR | TGTTCTTGCTGGAGCCGTTG |
| H-*SHP*-F | qRT-PCR | CCCAAGATGCTGTGACCTTT |
| H-*SHP*-R | qRT-PCR | CCAGAAGGACTCCAGACAGC |
| *18S*-F | qRT-PCR | AGTCCCTGCCCTTTGTACACA |
| *18S*-R | qRT-PCR | GATCCGAGGGCCTCACTAA |
| M-*36B4*-F | qRT-PCR | TGGAGACAAGGTGGGAGCC |
| M-*36B4*-R | qRT-PCR | CACAGACAATGCCAGGACGC |
| M-*IL-6*-F | qRT-PCR | CTTCTTGGGACTGATGCTGGT |
| M-*IL-6*-R | qRT-PCR | ACAGGTCTGTTGGGAGTGGTA |
| M-*IL-1β*-F | qRT-PCR | TGTTAGGCCATGAAGTC |
| M-*IL-1β*-R | qRT-PCR | GAAGTA AGGCAGAGAGC |
| M-*SHP*-F | qRT-PCR | CGATCCTCTTCAACCCAGATG |
| M-*SHP*-R | qRT-PCR | AGGGCTCCAAGACTTCACACA |
| M-*Sox9*-F | qRT-PCR | GGGATGCTGGCACAGAGAAT |
| M-*Sox9*-R | qRT-PCR | TGGCTCCTCTCGGATACCTC |
| M-*TNFα*-F | qRT-PCR | CATCTTCTCAAAATTCGAGTGACAA |
| M-*TNFα*-R | qRT-PCR | TGGGAGTAGACAAGGTACAACCC |
| M-*U6*-F | qRT-PCR | CGCTTCGGCAGCACATATACTA |
| M-*U6*-R | qRT-PCR | CGCTTCACGAATTTGCGTGTCA |
| M-*SHP*-ChIP-F | ChIP | CCCAACAAAAATCCATACTCGG |
| M-*SHP*-ChIP-R | ChIP | CCTTTTAGTCCTAGGAGTGGTTC |

H: Human M: Mouse F: Forward R: Reverse
